# Predictive assembling model reveals the self-adaptive elastic properties of lamellipodial actin networks for cell migration

**DOI:** 10.1038/s42003-020-01335-z

**Published:** 2020-10-26

**Authors:** Xindong Chen, Hanxing Zhu, XiQiao Feng, Xiaona Li, Yongtao Lu, Zuobin Wang, Yacine Rezgui

**Affiliations:** 1 School of Engineering, Cardiff University, Cardiff, CF24 3AA UK; 2 Institute of Biomechanics and Medical Engineering, AML, Department of Engineering Mechanics, Tsinghua University, 100084 Beijing, China; 3 School of Computer Science, Northwestern Polytechnical University, 710129 Xi’an, China; 4 State Key Laboratory of Structural Analysis for Industrial Equipment, Dalian University of Technology, 116024 Dalian, China; 5 International Research Centre for Nano Technology of China, Changchun University of Science and Technology, 130022 Changchun, China

**Keywords:** Biophysics, Motility, Cellular motility

## Abstract

Branched actin network supports cell migration through extracellular microenvironments. However, it is unknown how intracellular proteins adapt the elastic properties of the network to the highly varying extracellular resistance. Here we develop a three-dimensional assembling model to simulate the realistic self-assembling process of the network by encompassing intracellular proteins and their dynamic interactions. Combining this multiscale model with finite element method, we reveal that the network can not only sense the variation of extracellular resistance but also self-adapt its elastic properties through remodeling with intracellular proteins. Such resistance-adaptive elastic behaviours are versatile and essential in supporting cell migration through varying extracellular microenvironments. The bending deformation mechanism and anisotropic Poisson’s ratios determine why lamellipodia persistently evolve into sheet-like structures. Our predictions are confirmed by published experiments. The revealed self-adaptive elastic properties of the networks are also applicable to the endocytosis, phagocytosis, vesicle trafficking, intracellular pathogen transport and dendritic spine formation.

## Abstract (web summary)

Xindong Chen et al. simulate the sheet-like branched actin networks used by cells during cell migration using a novel 3D assembling model. They show how intracellular proteins regulate the network’s elastic properties and then affect cell migration, providing greater insight into the fundamental physical mechanisms of the previously published experimental observations.

## Introduction

**Fig. 1:**
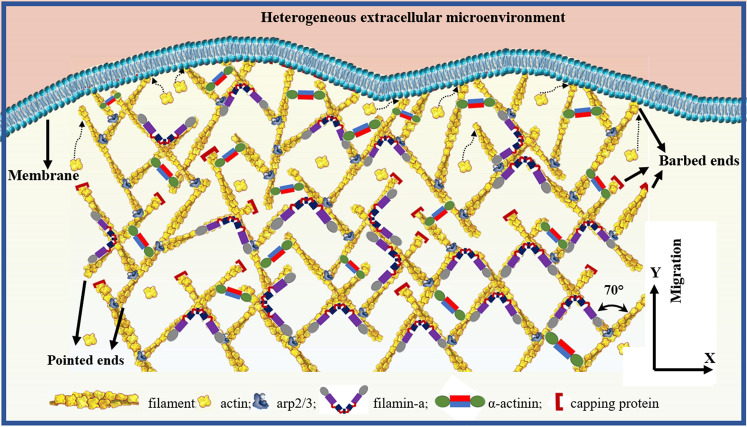
Branched actin network. Branched actin network structure in the front part of lamellipodium.

Cells are physical objects, which interact with extracellular microenvironments by generating, sensing, transmitting, and overcoming forces^1–4^. Cell migration based on lamellipodia, invadopodia, pseudopodia, and filopodia protrusions plays a crucial role in many physiological and pathological processes, e.g., cancer metastasis, embryonic morphogenesis, wound healing, tissue renewal, and autoimmune disorders^1,3,5^. Lamellipodia are sheet-like structures, and filopodia usually grow out from them^6^. For lamellipodia-based cell migration, lamellipodial branched actin network not only generates a pushing force by actin polymerization but also provides crucial mechanical support for cell migration through the extracellular matrix or adjacent cells^1,7,8^. Arp2/3 complex nucleates new filaments by an angle of ~70° from pre-existing filaments and creates dendritic subnetworks^9^. These dendritic subnetworks are cross-linked together by filamin-A and α-actinin, forming an interconnected branched actin network (Fig. 1)^1,10,11^. In vivo, for both single cell and collective cells, their migrations based on lamellipodia are largely determined by the mechanical interactions between the lamellipodial branched actin networks and the confining extracellular microenvironments^1,8,12,13^. During migrations, cells seldom experience mechanically isotropic microenvironments^8,14,15^. Thus, cell migration is more like an active cellular self-adaptive behavior^1,8,16^. Although extensive experimental studies have been conducted, the intracellular self-regulation mechanisms of migratory cells based on lamellipodia overcoming varying extracellular microenvironment have not been deciphered. A comprehensive and clear description of these mechanisms is of great significance for exploiting effective therapies for diseases associated with abnormal cell migrations^17–19^.

A major challenge to investigate the mechanical properties of the sheet-like branched actin network is that it is in a highly dynamic and stochastic remodeling state with mechanochemical interactions of intracellular proteins, such as nucleating, actin polymerizing and depolymerizing, Arp2/3 complex branching, capping protein-inhibiting polymerization, cross-linking proteins binding and unbinding^7,20,21^. The networks’ complexity and stochastic biological process hinder one from performing an adequate number of biological experiments or carrying out mechanical analysis to study the quantitative relationships between the macroscopic elastic properties and the microscopic structures regulated by various intracellular proteins^22^. To identify such relationships, probing the kinetic architecture and measuring its corresponding mechanical response should be done simultaneously. Moreover, the properties of the branched actin network contain several independent elastic parameters (“Methods”, Eq. (34)). To decipher the mechanisms of how migrating cells overcome 3D extracellular confinements, it is essential to obtain sufficient mechanical parameters of the assembling branched actin networks and thereby to analyze their impacts on cell migration by considering extracellular resistance. However, such work is still lacking.

In addition, recent experiments revealed that the orientation distribution of actin filaments in the branched actin network is regulated by different magnitudes of extracellular resistance^8^. Given that the branched actin network provides essential mechanical support for cell migration, such mechanosensitive orientation changes may be related to the mechanical adaptability for cell migration. However, the underlying physical mechanism of the architecture transitions induced by different extracellular resistances remains an open question.

Recently, biological scientists jointly appeal for building predictive spatiotemporal cell models to open new dimensions in biological research^23^. Constructing predictive models at the intersection of biology, mathematics, physics, and computer science is an effective way to perform quantitative analysis and elucidate the underlying mechanisms of complicated biological questions^23–25^. In this paper, a three-dimensional self-assembling model of lamellipodial branched actin network during cell migration is constructed by taking into account of five types of key proteins, i.e., filamentous actin (F-actin), Arp2/3 complex, capping protein, filamin-A and α-actinin, and their assembling interactions, e.g., filament polymerizing, Arp2/3 complex branching, capping protein-inhibiting polymerization, and actin cross-linking proteins’ binding and unbinding. Then, the network is simplified to be linear elastic, although the real structure shows viscoelastic behaviors^1,26^. Combining this multiscale assembling model with the finite element method (FEM), we have studied more than 4000 stochastic models of the lamellipodial actin network during cell migration. These results allow us to capture the underlying physical mechanism of the experimentally observed puzzles about the self-adaptive behaviors of the lamellipodial actin network in response to varying extracellular resistance^1,8^. In addition, by probing the microscopic self-assembling architecture remodeled by intracellular proteins and the macroscopic mechanical responses simultaneously, we quantitatively identify how these intracellular proteins respectively or in combination regulate the elastic properties of the branched actin network on macromolecular levels. The results well predict and explain the experimental observations about the impacts of F-actin, Arp2/3 complex, filamin-A and α-actinin on migrating cell leading-edge behaviors^1,7,8,10,15,27–33^. More importantly, combined with the published experimental findings^34^, this study reveals the intracellular self-adaptive physical mechanisms of the migratory cell leading edges in response to varying extracellular resistance during cell migration. Such a self-adaptive physical mechanism of branched actin network may apply to endocytosis, phagocytosis, vesicle trafficking, intracellular pathogen transport, and dendritic spine neurodevelopmental^35–37^.

## Results

### The adaptive model simulates the assembling process of the lamellipodial branched actin network during cell migration

**Fig. 2:**
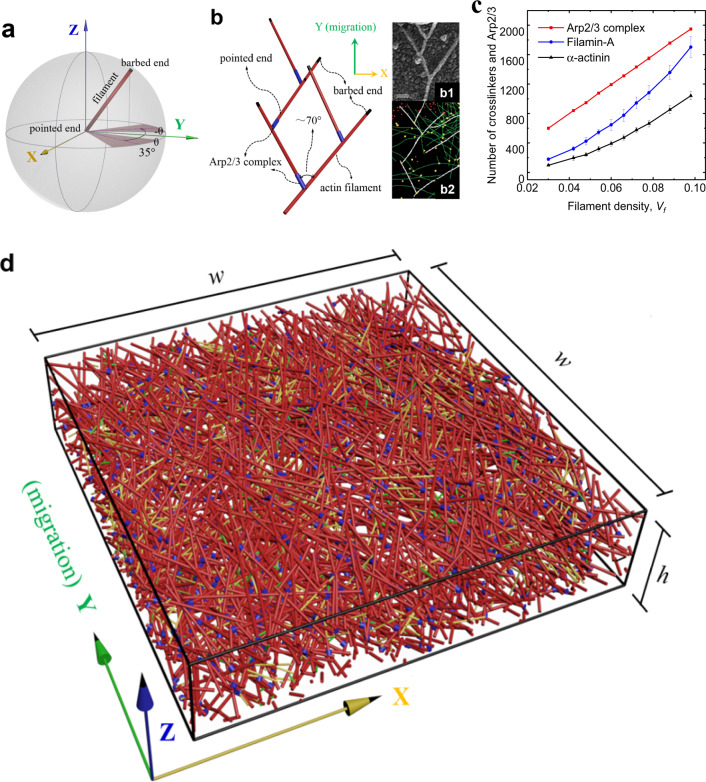
Construction process of the dynamic branched actin network model and its validation. **a** Stochastically created actin filaments with barbed end polymerizing forward based on the spherical coordinate system (shadow areas are the preferential angle with respect to the cell moving direction). **b** The dendritic structure created by the Arp2/3 complex nucleating and branching out from existing filaments stochastically in our model; the inserted figures (b1) and (b2) are fluorescence microscopic images of branched actin filament from refs. ^36^ and ^13^, respectively. Each data point in our results is a mean value calculated from about 30 stochastic models with the same set of parameters (Supplementary Tables 2–4). **c** Numbers of Arp2/3 complex, filamin-A and α-actinin per µm^2^ in the *xy* plane of the models. **d** A representative volume element (RVE) model of the branched actin network (red: actin filament; blue: Arp2/3 complex; yellow: filamin-A; green: α-actinin). This model is periodic in the *xy* plane. Its side lengths in both the *x* and *y* directions are *w* = 1000 nm and thickness in the *z* direction is *h* = 200 nm, which is a typical thickness of lamellipodia. The *x*, *y*, and *z* axes are along the transverse direction, cell migration direction, and out-of-plane direction, respectively.

In order to create the predictive assembling model of the highly dynamic branched actin network, we first simulate its realistic stochastic self-assembling process in the sheet-like lamellipodial space during cell migration. In this mathematical model, a number of realistic parameters, five types of key proteins in lamellipodia, i.e., F-actin, Arp2/3 complex, capping proteins, filamin-A and α-actinin, and their mechanochemical interactions are considered. Briefly, in the sheet-like lamellipodial space, by referring to F-actin concentration, mother filaments are first generated with preferred orientations with respect to cell migration direction (Fig. 2a). In lamellipodia, the polymerization and depolymerization rates of actin are in a dynamic steady state. Thus, we only consider the net polymerization (“Methods”, Eq. (1)). The ultimate growth length of actin filaments is generated through a Gaussian distribution based on published experimental data^32,35,38^. Arp2/3 complex can randomly bind on actin filaments generating nucleation cores with reasonable intervals and create branches. Due to the particular branch angle of about 70° formed by the Arp2/3 complex, the possible position of the Arp2/3 complex branch is on a circular conical surface around the mother filament. To be consistent with in vivo condition, the randomly selected Arp2/3 complex branches from the conical surface should meet the orientation requirement relative to the cell migration direction. At the same time, it should allow daughter filaments to polymerize to a reasonable length in the lamellipodial sheet-like space. Then, daughter actin filaments begin to grow out from the Arp2/3 complex branches and are capped by capping proteins when they reach their growth lengths. In a similar way, available Arp2/3 complex binds on the daughter filaments and nucleates a new generation of daughter filaments. After several time steps, dendritic structures are generated (Fig. 2b), and the total length of the branched actin filaments is determined by F-actin concentration. In the assembling process of the branched actin filaments, cross-linking proteins, i.e., filamin-A and α-actinin, are also generated to bind on and crosslink them. Instead of liking the actin cortex model^39^ where a crosslinker is generated only according to the shortest distance between two filaments and there is only one crosslinker between two filaments, we generate cross-linking proteins according to the relative orientations and distance of the two actin filaments in the three-dimensional sheet-like space, which is more consistent with the intracellular condition. Additionally, like the true condition in cells, two actin filaments in our model can be cross-linked by several cross-linking proteins with appropriate intervals. Finally, our mathematical model is validated by replicating the dendritic architecture of the branched actin network (Fig. 2b) and by predicting the densities of filamin-A and α-actinin relative to that of the Arp2/3 complex in migrating lamellipodia where the density of the Arp2/3 complex is larger than that of filamin-A, and the latter in turn is larger than that of α-actinin in the branched actin network (Fig. 2c)^11^. In addition, the predicted number of filaments at cell migration leading edge is also consistent with the experimental measurements (“Methods”, Model validation). A detailed description of the model is in “Methods”. Note that, with our mathematical model, the in vivo microscopic spatial dynamic variation and reconfigurability of lamellipodial branched actin network, which are regulated by different intracellular proteins and variable confining extracellular microenvironments during cell migration, can be carefully simulated by regulating F-actin concentration, actin filament-polymerizing orientation, Arp2/3 complex successive branching, Arp2/3 complex branching density, and cross-linking proteins (filamin-A and α-actinin) binding and unbinding, respectively or combinedly. Using this mathematical model, we construct continuum mechanics-based three-dimensional regulatable representative volume element (RVE) models (Fig. 2d) of the branched actin network by assigning the experimentally measured geometric and elastic properties of the actin filament, Arp2/3 complex, filamin-A and α-actinin materials, and periodic boundary conditions. Then, the elastic properties and self-adaptive mechanism of the highly remodeling lamellipodial branched actin network driving cell migration through varying extracellular microenvironments are investigated.

### Resistance-adaptive actin filament density improves the network stiffness sensitively

During cell migration, actin filament assembly and disassembly occur simultaneously in the lamellipodial branched actin network, which makes the network in a perturbation state^20^. Actin filament density *V*_*f*_ is defined as the volume fraction of actin filaments in the sheet-like lamellipodial space (Supplementary information, Eq. (S1)). It is normally in the range of 3.0–10%^26,40–43^ and is correlated to F-actin concentration (“Methods”, Eqs. (2)). Experimental results show that when the confining extracellular resistance increases, filament density in the branched actin network also increases^1,8,28^. Here, we investigate why filament density fluctuates with extracellular resistance, how it regulates the elastic properties of the lamellipodial actin network and how the latter, in turn, affects cell protrusion in highly heterogeneous 3D extracellular microenvironments.

**Fig. 3:**
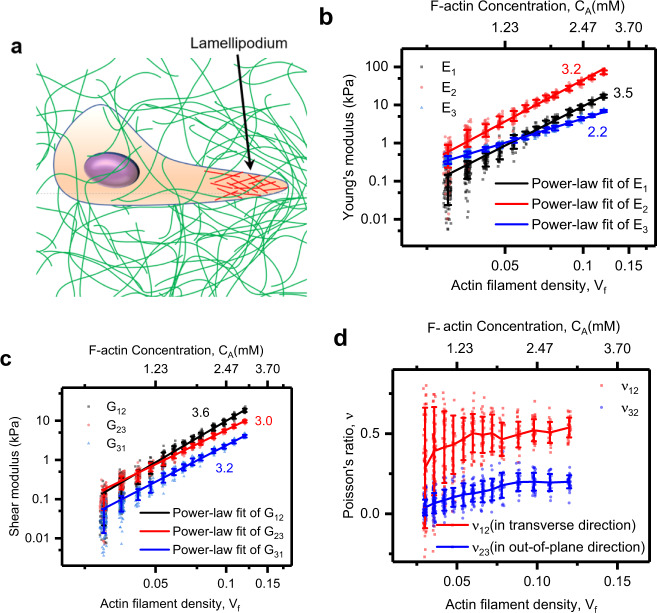
Actin filament density (or F-actin concentration) sensitively regulates the elastic properties of the branched actin network. **a** lamellipodium drives cell migration through confining extracellular microenvironments. **b** Young’s moduli: *E*_1_, *E*_2_, and *E*_3_ are in the transverse direction (*x* direction in Fig. 2c), cell movement direction (*y* direction in Fig. 2c), and the out-of-plane direction (*z* direction in Fig. 2c), respectively. **c** Shear moduli: *G*_12_ is in the *xy* plane, *G*_23_ is in the *yz* plane, and *G*_31_ is in the *xz* plane. **d** Poisson’s ratios are defined as $v_{ij} = - \varepsilon _i/\varepsilon _j$, where $\varepsilon _i$ is the strain in the *i* direction when uniaxial stress is applied in the *j* direction. The data in figures for power-law fit are mean values calculated from about 30 stochastic models with the same set of parameters of intracellular proteins, and the error bars are the standard deviations of the results. The numbers near the straight fitting lines in panels **b** and **c** are the slopes of these power-law fit functions.

Our results show that both the Young’s and shear moduli of the branched actin network scale with F-actin concentration *C*_*A*_ (or filament density *V*_*f*_): $E_1 \sim C_A^{3.5}$, $E_2 \sim C_A^{3.2}$, $E_3 \sim C_A^{2.2}$, $G_{12} \sim C_A^{3.6}$, $G_{23} \sim C_A^{3.0}$, and $G_{31} \sim C_A^{3.2}$ (Fig. 3b, c). The scaling exponents of the branched actin network are much larger than those of the cross-linked actin network, e.g., $C_A^{2.0 \sim 2.5}$^1,44^. Thus, compared with the cross-linked actin network, the stiffness of the branched actin network is much more sensitive to filament density. Young’s modulus *E*_2_ is always much larger than *E*_1_ and *E*_3_, indicating that the network is highly anisotropic and the stiffness in the cell movement direction is the largest. This is important for cell migration because insufficient stiffness in the migration direction of the branched actin network is unable to overcome the confining resistance and thus may cause the cell to lose mobility^15^. Our prediction also well interprets the directional actin-based motility that the overall direction of branched actin network growth is deflected toward denser area^28^. In addition, our results are in good quantitative agreement with both the in vivo and in vitro experimental data (Supplementary Table 5) in refs. ^1,26,31,45–47^. For example, the filament density *V*_*f*_ of the branched actin network in keratocyte lamellipodium is normally ~8%^48^, and it is in vivo measured Young’s modulus *E*_2_ is in the range of 21–44 kPa^46^, which agrees well with our numerical prediction: 16–39 kPa when the filament density is from 7.0% to 9.8%. Even though the exact filament densities in these published experiments are not given, our results are in the same order with these experimental data, especially in contrast with the over 100-fold magnitude difference of the cross-linked actin network between previous studies and in living cells^49^.

Among the shear moduli, the shear modulus *G*_12_ in the migration plane is the largest. It scales strongly with actin filament density: $G_{12} \sim C_A^{3.6}$. A recent experiment showed that heterogeneity in the branched actin network is a dominant factor for steering cell movement^28^. Moreover, filopodia usually grow out from the branched actin network in cancer cells to protrude forward^12^. They both indicate that the branched actin network must be able to bear high shear force in the moving plane since the active moving area or invadopodia growing area undertakes a much higher load than other areas. Thus, the high shear modulus *G*_12_ is important for maintaining the stability of the branched actin network in the cell-directional migration process.

To explore whether the actin filaments or the cross-linking proteins dominate the stiffness of the branched actin network, we perform some separate finite element numerical simulations/tests by using Young’s modulus ten times larger or smaller than the actual *E*_*f*_ of actin filaments or the actual *E*_*c*_ of the cross-linking proteins, and find that the stiffness of the branched filament networks is primarily dependent on the stiffness of the actin filaments and less sensitive to the stiffness of the cross-linking proteins (Supplementary Fig. 2). Moreover, the gradient of the log-log scaling relationship between Young’s modulus *E*_2_ of the branched filament network and the actin filament density is larger than three (Fig. 3b), indicating that the bending deformation of actin filaments is the dominant deformation mechanism of the branched filament networks. Additionally, under uniaxial compression tests in the cell migration direction, both Poisson’s ratios *v*_12_ and *v*_32_ increase with increasing filament density (Fig. 3d). Strikingly, *v*_12_ is always much larger than *v*_32_, which suggests that the network is much easier to deform in the in-plane transverse direction rather than the out-of-plane direction (i.e., the thickness). Collectively, they indicate that when cell migrates under extracellular resistance, the deformation of the branched actin network is predominately the backward bending of actin filaments in the migration plane. Because the Arp2/3 complex is preferential to binding on the convex side of a bent mother filament and branching out a daughter filament^34^, the results explain why lamellipodium grows into a sheet-like structure and protrudes forward. More importantly, they also reveal the physical mechanism of the recent experimental finding that a high extracellular resistance induces a high filament density in lamellipodia^8^: if the stiffness of the lamellipodial branched actin network with a low filament density is not sufficient to overcome the confining extracellular microenvironment, actin filaments in it will be largely bent in the migration plane, and thus more Arp2/3 complex will bind on the convex side of the bent filaments to branch out more daughter filaments making the filament density increase, which in turn sensitively strengthens the network to overcome the extracellular resistance and propel cell migration.

### Successive branches formed by the Arp2/3 complex are essential for cell migration

**Fig. 4:**
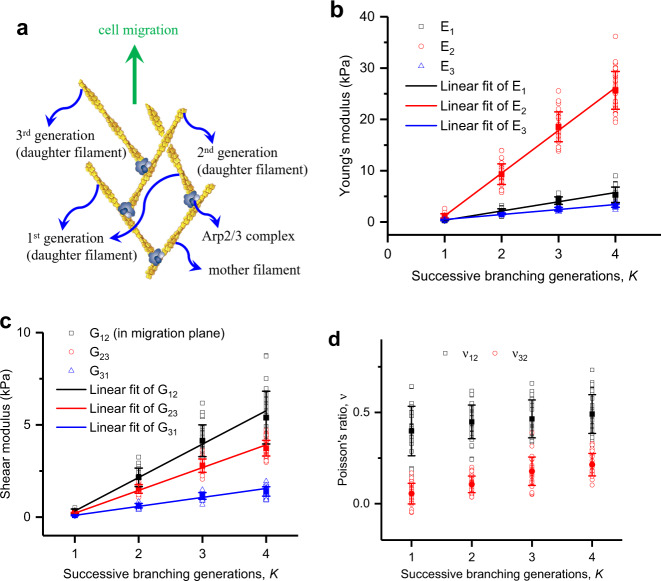
The number of successive branching generations from a mother filament regulates the elastic properties of the branched actin network. **a** Successive branching generations in dendritic structure. **b** Young’s moduli. **c** Shear moduli. **d** Poisson’s ratios under the compressive force along the movement direction. The solid data points in figures are mean values calculated from about 30 stochastic models with the same set of parameters of intracellular proteins, and the error bars are the standard deviations of the results.

In this section, we explore the effect of the successive branching generation number *K* created by Arp2/3 complex from mother filaments (Fig. 4a) on regulating the elastic properties of the network, and then investigate its possible value by calculating the network deformation under actin filament propulsive force. Here, we take the lamellipodium of keratocytes as an example, whose filament density is ~7.8%. Our results show that the Young’s and shear moduli approximately linearly increase with the successive branching generation number *K* (Fig. 4b, c). This sensitive enhancement effect on the network stiffness can be interpreted by the increase in the relatively rigid dendritic size (Fig. 4a) in the migration direction. We then ask whether the branched actin network with a small number of successive branching generations *K* is able to support cell migration or not. In keratocyte lamellipodium, each filament averagely produces a pushing force of about 2 pN by polymerization^50^, and about 150 filaments are pushing against per µm length of the leading membrane^43,51^. Thus, the stress under the resultant pushing force in this direction can be calculated as 1.5 kPa (Supplementary information, Eq. (S2)). This indicates that the compressive strain of the branched actin network with *K* = 2 (Fig. 4b) is >15% (Supplementary Information, Eq. (S3)), implying that the network would be too soft and thus can not effectively support the pushing force for cell motility. Therefore, we conclude that most of the subnetworks should have at least three successive branching generations in a protruding lamellipodium. Our prediction is supported by the high-resolution experimental images showing that filaments in migrating lamellipodia branch in several successive generations^7,11,36^. Because the Arp2/3 complex prefers to bind on bent filaments^34,52^ and thus the branched actin network can regulate its successive branches to adapt for cell migration, the low number of successive branching generations in experiment^13^ might be observed from cells that were not in an active migration state.

### Strengthening and local heterogeneous weakening effects of self-regulated Arp2/3 complex density on the network stiffness

Now we examine the effects of the Arp2/3 complex branching density *n*_*arp*_ on the elastic properties of the branched actin network. To avoid the influence of successive branches formed by Arp2/3 complex, we deliberately regulate the model and control the number of successive branching generations as *K* = 3. The density of Arp2/3 complex *n*_*arp*_ is defined as its average number along the average length of actin filaments (Supplementary information, Eq. (S4)). Because the branching connection formed by Arp2/3 complex is relatively rigid^53^, the average value of *d*_*arp*_, defined as the distance between two adjacent Arp2/3 complexes along a filament, is also named as the characteristic length *l*_*c*_ in the branched actin filament^31^.

**Fig. 5:**
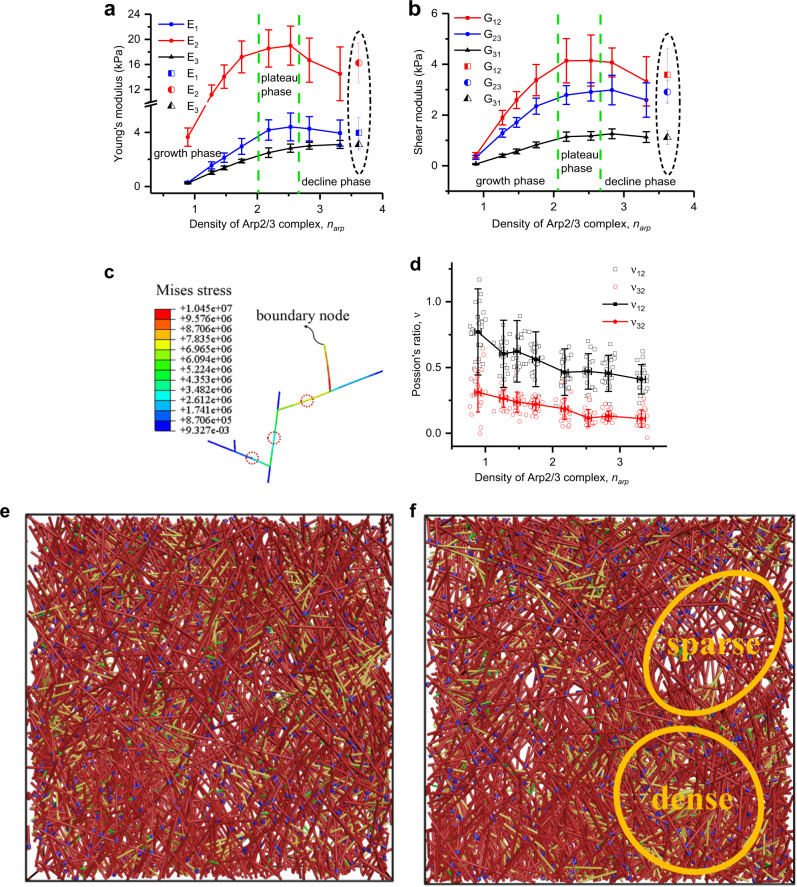
Arp2/3 complex density *n*_*arp*_ regulates the elastic properties of the branched actin network. **a** Young’s moduli. **b** Shear moduli. The green dashed lines in **a** and **b** divide the curves into three stages: growth phase, plateau phase, and decline phase. The results in the dashed ellipses are obtained from the models with a more homogeneous distribution of Arp2/3 complex density *n*_*arp*_. **c** von Mises stress distribution in the local structure of the network. **d** Poisson’s ratios. **e** Heterogeneity of branched actin networks ($\Omega = 18.4\%$) when Arp2/3 complex density *n*_*arp*_ = 2.5 and filament density is 7.8%. **f** Heterogeneity of branched actin networks ($\Omega = 39.2\%$) when Arp2/3 complex density *n*_*arp*_ = 3.3 and filament density is 7.8%. Each solid data point in figures is an average value calculated from about 30 stochastic models with the same set of parameters of intracellular proteins, and the error bar is the standard deviation of the results.

The results show that the evolution of both the Young’s and shear moduli with the increase of branching density is triphasic, i.e., roughly linear growth phase, plateau phase, and decline phase (Fig. 5a, b). In the first phase, when the branching density *n*_*arp*_ increases in the normal range from 0.9 to 2.2, it has a noticeable improving effect on the elastic properties of the branched actin network, especially on *E*_1_, *E*_2_, and *G*_12_ in the cell migration plane (Fig. 5a, b). These results explain the experimental findings^29,30,54^ that inhibition of the Arp2/3 complex for actin nucleation negatively regulates cell migration and invasion. We interpret this sensitive relationship between the macroscopic elastic properties and microscopic branching density formed by the Arp2/3 complex as the result of the decrease in the characteristic length *lc*. As demonstrated by the stress contour along branched filaments (Fig. 5c), stress mainly distributes in the filament segments formed by two adjacent Arp2/3 complex branch points. Then, when the branching density is between 2.2 and 2.5, the evolution of these Young’s and shear moduli step into a plateau phase (i.e., the second phase) where the variation of branching density has a little impact on the network stiffness due to saturation.

However, when the branching density is ~2.5 (i.e., the third phase), its further increase unexpectedly lowers all the Young’s and shear moduli. This indicates that under a constant F-actin concentration when the branching density is too high, the stiffness of the branched actin network decreases, which is inefficient to support cell migrations. Experimental results also show that excessive high branch density formed by the Arp2/3 complex leads to slower cell lamellipodium leading-edge protrusion^32^. To further investigate the physical mechanisms of why an excessive high branching density induces a lower mechanical stiffness, we check the architectures of these self-assembling models. The RVE model is divided into nine equal parts in the *xy* plane, and then the density of actin filaments in each part is calculated. We define the network heterogeneity Ω (Supplementary Information, Eq. (S5)) as the coefficient of density variation, which is the standard deviation of the densities in the nine parts over their average density. Strikingly, the actin network with high Arp2/3 branching density shows severe local heterogeneity Ω = 39.2% (Fig. 5e, f). Because the generation of daughter filaments is controlled by Arp2/3 complex branching, excessive branching of Arp2/3 complex inevitably results in local heterogeneity of the global network under a constant F-actin concentration. We then deliberately regulate the stochastic generation process of the Arp2/3 complex to make the distribution of the branches more homogeneous. Our simulation results (highlighted by dashed ellipses in Fig. 5a, b) indicate that although the density of the Arp2/3 complex *n*_*arp*_ increases to 3.62, the Young’s and shear moduli are both improved. Consequently, it is the network heterogeneity induced by excessive high branching of Arp2/3 complex that causes the low elastic stiffness. This is also confirmed by the experimental observation that local fractures in the branched actin network occur under the resistance load for cell motility^33^. However, cells have self-regulation mechanisms to optimize their branching density to favor their movements. For example, Profilin, Ena/VASP, Arpin, and Gadkin proteins in lamellipodia can negatively regulate the density of the Arp2/3 complex branches^20,32,55^. Heterogeneity resulted from high branching density of the Arp2/3 complex; however, it is not always adverse to cell migrations. It is an important way for cells to steer their migration directions^28^.

Our results show that for the branched actin network with the normal filament density of 7.8%, its stiffness reaches the peak value when the branching density is about 2.5, indicating that the optimal spacing between two adjacent branching points along a filament is ~100 nm. In addition, both the Poisson’s ratios *v*_12_ and *v*_32_ noticeably decrease with increasing branching density, and the in-plane *v*_12_ is also always larger than the out-of-plane *v*_32_.

### Density of cross-linking proteins regulated by filament density linearly strengthens the network stiffness by increasing connectivity

**Fig. 6:**
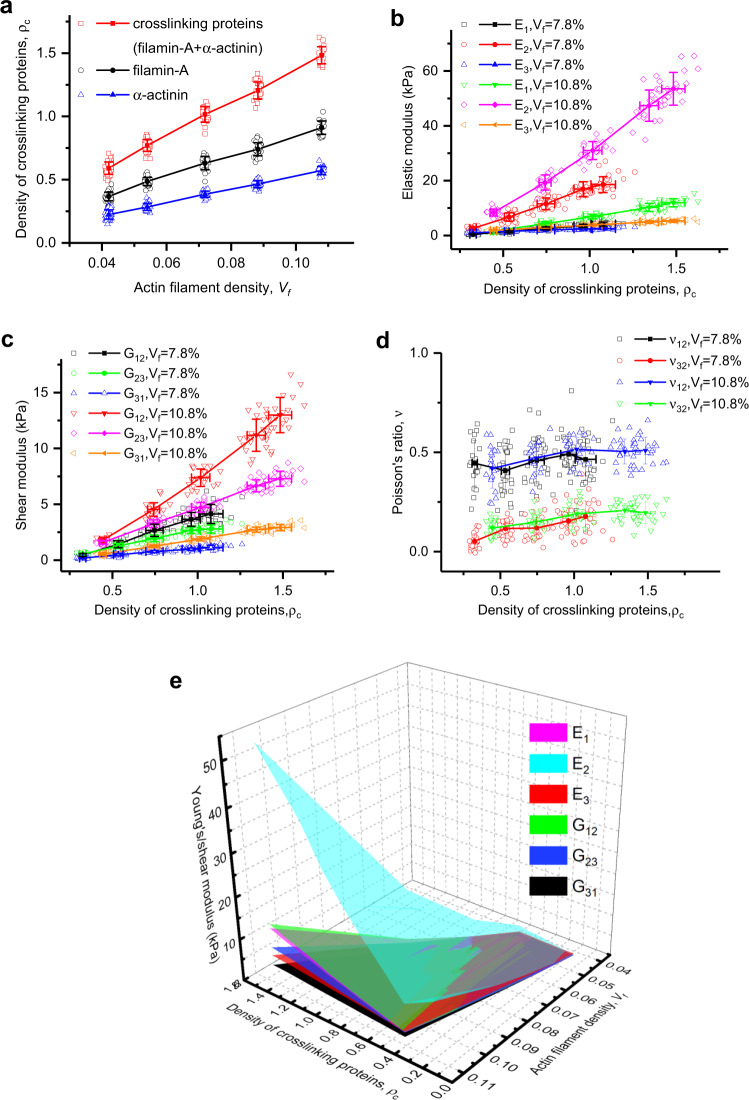
Maximum density of cross-linking proteins versus filament density, and the relationships between the cross-linking protein density and the elastic properties of the branched actin network. **a** Maximum density of cross-linking proteins versus densities of actin filaments. **b** Young’s modulus versus the density of cross-linking proteins. **c** Shear modulus versus the density of cross-linking proteins. **d** Poisson’s ratios under uniaxial stress in the *y* axis versus the density of cross-linking proteins. **e** Comparison of Young’s and shear moduli obtained from 15,000 numerical simulations for >2400 stochastic models under different combinations of filament densities and cross-linking densities. It shows that Young’s modulus *E*_2_ in the cell movement direction is much larger than the others. Each solid data point in figures is an average value calculated from about 30 stochastic models with the same set of parameters of intracellular proteins, and the error bar is the standard deviation of the results.

Mutations and dysfunction of cross-linking proteins affect the mechanical performance of cross-linked actin networks and thus lead to diseases^56,57^. Here, we ask how their binding and unbinding influence the elastic properties of the lamellipodial branched actin network, and then how they affect cell migration. We define the cross-linking protein density *ρ*_*c*_ (filamin-A and α-actinin) as their average number along the average length of a filament (Supplementary Information, Eq. (S6)). The maximum cross-linking density *ρ*_*c*_ is found to be proportional to the filament density (Fig. 6a and Supplementary Fig. 3), suggesting that the density of cross-linking proteins can be regulated by filament density.

Analogously to the impact of the number of successive branching generations, all the Young’s and shear moduli increase linearly with the cross-linking protein density (Fig. 6b, c). For the common filament density of 7.8% in keratocytes, as the cross-linking protein density increases from 0.33 to 1.08, Young’s moduli *E*_1_, *E*_2_, and *E*_3_ increase from 0.48 to 4.17 kPa, 2.60 to 18.55 kPa, and 0.99 to 2.50 kPa; and shear moduli *G*_12_, *G*_23_, and *G*_31_ rise from, 0.46 to 4.14 kPa, 0.54 to 2.79 kPa, and 0.18 to 1.15 kPa, respectively. The improving effects on *E*_2_, *E*_1_, and *G*_12_ in the lamellipodium protrusion plane are very prominent (Fig. 6b–e). In addition, Young’s modulus *E*_2_ is improved from 2.60 to 18.55 kPa, which agrees well with the experimental results^1^ that cross-linking proteins, i.e., filamin-A and α-actinin, enhance *E*_2_ of the branched actin network from 6 kPa to ~20 kPa. Comparison of the curve gradients at different filament densities reveals that with the increase in the filament density, the cross-linking protein density exhibits a more distinct influence on the stiffness of the branched actin network (Fig. 6b–e). Poisson’s ratios *v*_2_ and *v*_23_ slightly increase with the increase in the cross-linking protein density (Fig. 6d) as a result of increased connectivity in the network. Moreover, in all simulations, Young’s modulus *E*_2_ is significantly larger than the other elastic moduli (Fig. 6e).

Although the cross-linking proteins are very flexible^58–60^, their stabilizing effect by increasing the interpenetrating connectivity in the branched actin network is rather distinct. The branched actin network with a low density of cross-linking proteins is incapable of supporting the propulsion force for cell motility. This reveals the underlying physical mechanism for the experimental finding of human melanoma cells that without cross-linking protein filamin-A, individual Arp2/3 complex is insufficient for maintaining the mechanical stability of the branched actin network at the leading edge^10^. More importantly, we find that the cross-linking protein density has a linear relation with the filament density. Increasing extracellular resistance can induce an increase of filament density during cell migration^1,8^. Consequently, increasing resistance can induce the assembling of cross-linking proteins in the branched actin network, which in turn makes the network stiffer to adapt to the increased resistance for cell migration.

### Resistance-adaptive filament orientation transitions tend to meet the stiffness demand for cell migration

**Fig. 7:**
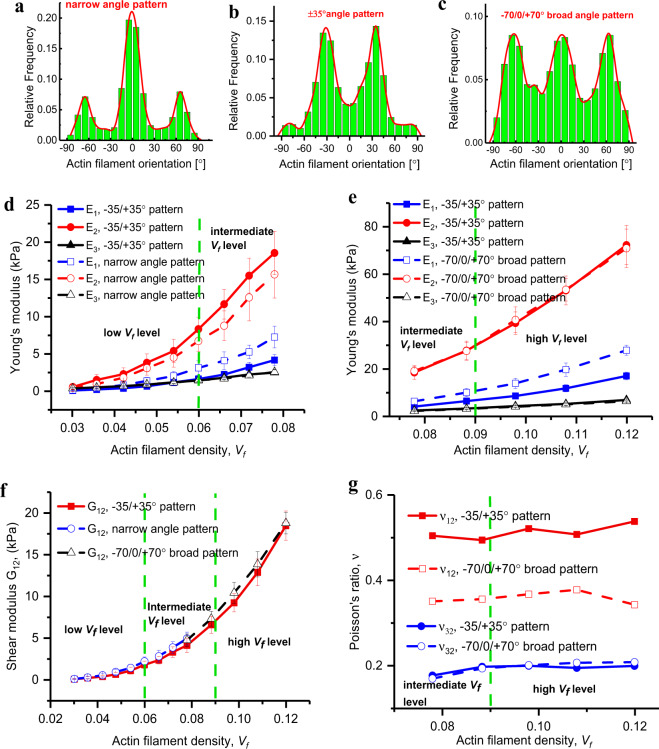
Actin filament orientation transitions regulate the network elasticity. **a** Narrow-angle pattern of actin filament orientation. **b** ±35° angle pattern of actin filament orientation. **c** −70/0/+70° broad-angle pattern of actin filament orientation. **d** Comparison of Young’s moduli between the narrow-angle pattern and the ±35° pattern. **e** Comparison of the Young’s moduli between the ±35° pattern and the −70/0/+70° broad-angle pattern. **f** Comparison of the shear moduli between the narrow-angle pattern, the ±35° pattern, and the −70/0/+70° broad-angle pattern. **g** Comparison of the Poisson’s ratios between the ±35° pattern and the −70/0/+70° broad-angle pattern. The green dashed lines in **d**, **e**, **f**, and **g** divide the actin filament density into three levels: low level, intermediate level, and high level. Data in the figures are an average value calculated from about 30 stochastic models with the same set of parameters of intracellular proteins, and the error bar is the standard deviation of the results.

The orientation of actin filaments in the branched actin network, defined as the angle between an actin filament and the cell migration direction, is an important characteristic presented during cell mobility^8,51,61,62^. Both experimental^8^ and simulation^62^ studies show that actin filaments in the branched actin network exhibit three types of orientation distribution patterns, i.e., narrow-angle pattern (Fig. 7a), ±35° angle pattern (Fig. 7b), and broad-angle pattern (Fig. 7c)^61–64^. As the extracellular resistance load increases from low to high, the orientation distribution of actin filaments in the branched actin network transforms from the narrow-angle pattern to the ±35° angle pattern and then to the broad-angle pattern, meanwhile, cell migration velocity decreases^8,62^. Here, we construct the three types of architecture models of the branched actin networks (Fig. 7a–c) and explore the underlying physical mechanism of their architecture transformations in response to the variation of extracellular confining resistance.

When the filament density is low (<6.0%), the filament network with the narrow-angle pattern has larger Young’s modulus *E*_1_ and shear modulus *G*_12_, and similar Young’s moduli *E*_2_ and *E*_3_ compared to those of the network with the ±35° pattern (Fig. 7d, f). This indicates that the narrow-angle pattern network with a low filament density is overall stiffer than its counterpart network with the ±35° pattern. However, when the filament density increases to intermediate level, Young’s modulus *E*_2_ in the cell movement direction of the ±35° pattern network exceeds that of the narrow-angle pattern network, suggesting that the ±35° pattern filament network is more stable and effective in supporting cell migration. Since the increase in the filament density is induced by increasing extracellular resistance^8^, this helps to explain the experimental results that with the increase of extracellular confining resistance, the network architecture transforms from the narrow-angle pattern to the ±35° pattern. More specifically, in the initial stage of migration, the cell is subject to a low resisting force, and most of the filaments grow perpendicularly to the leading membrane; thus an efficient pushing force can be generated to drive cell forward with rapid velocity. However, with the increase in the resistance force, the filament network with the narrow-angle pattern cannot support its migration in this direction. Consequently, the branched actin filaments are bent and rotated under the force (meanwhile, because the Arp2/3 complex prefers to bind on the bent filaments, this also improves the possibility of the Arp2/3 complex nucleating more daughter filaments and makes the network’s filament density increase from the low level to the intermediate level), changing their orientations into the ±35° pattern to meet the stiffness demand in the cell migration direction.

When the filament density *V*_*f*_ increases to a higher level (>9.0%), the Young’s modulus *E*_1_ and shear modulus *G*_12_ of the −70/0/+70° broad-angle pattern network are much larger than those of the ±35° pattern network, while the Young’s moduli *E*_2_ and *E*_3_ of the two patterns are almost the same (Fig. 7e, f). In addition, compared to the Poisson’s ratio of the ±35° pattern network, the Poisson’s ratio *v*_12_ of the −70/0/+70° broad-angle pattern network is much smaller. These results consistently indicate that the −70/0/+70° broad pattern filament network has stiffer mechanical properties. This provides an explanation for the experimentally observed secondary transformation^8^ that when the extracellular confining resistance increases from intermediate range to high range, the filament network architecture transforms from the ±35° pattern into the broad-angle pattern. More specifically, with the increase in the resistance force, the stiffness of the filament network with the ±35° pattern is incapable of overcoming the extracellular resistance force. Thus, the filaments rotate and grow denser, leading to the network architecture transforming from the ±35° pattern into the −70/0/+70° broad-angle pattern. We speculate that, under a high extracellular-resistance load, the branched actin network needs higher *E*_1_ and *G*_12_ to prevent large transverse and shear deformations in the migration plane, and consequently its network architecture is adjusted to meet the stiffness demand for cell migration.

## Discussion

### Resistance-adaptive elastic properties of branched actin network remodeling with intracellular proteins and altering geometry

**Fig. 8:**
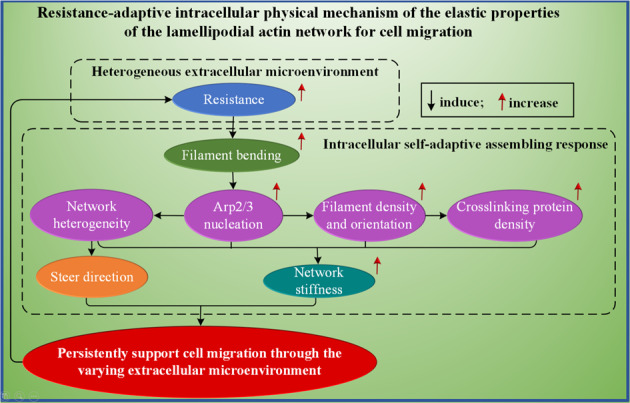
Intracellular biophysical mechanisms. Resistance-adaptive intracellular physical mechanism of the elastic properties of the lamellipodial branched actin network for cell migration in heterogeneous extracellular microenvironment.

The three-dimensional extracellular microenvironments are usually extremely complex and mechanically heterogeneous^1,8,14,15^. When the lamellipodial branched actin network supports a cell migrating through them, it experiences highly varying immediate resistance^1,8,14,28^. On a macromolecular level, we propose a possible fundamental biophysical mechanism that migratory cells with lamellipodia mechanically sense and adapt to the heterogeneous extracellular confining microenvironment (Fig. 8). To be specific, the deformation mechanism of the lamellipodial branched actin network is mainly dominated by the bending deformation of actin filaments. Because the Arp2/3 complex prefers to bind on the convex side of a bent actin filament and nucleates a daughter actin filament^34^, when extracellular resistance increases, the actin filaments in the branched actin network near the leading edge will be bent more severely, which triggers the mechanochemical reaction of the Arp2/3 complex. Thus, more Arp2/3 complexes will bind to them and nucleate more daughter filaments. Moreover, the increased actin filaments improve the cross-linking density of cross-linking proteins in the branched actin network (Fig. 5a). This indicates that the assembly of cross-linking proteins in the branched actin network is also a resistance-adaptive behavior. Combinedly, the stiffness of the lamellipodial branched actin network will be sensitively enhanced by the increased assembling densities of actin filaments, Arp2/3 complex, filamin-A, and α-actinin (Figs. 2–5). Furthermore, our study unveils the physical mechanism underlying filament orientation transitions induced by increasing resistance. Each transition makes the branched actin network stiffer, which suggests that the transitions are also mechanical adaptation behaviors for cells to overcome the confining resistance. These structural changes are attributed to the kinetic properties of Arp2/3 and the mechanical interaction between the actin filaments and the extracellular resistance acting on the lamellipodial leading-edge membrane. Experimental results show that when the membrane tension is low (due to low extracellular resistance), the filaments at small angles have a higher rate of survival^8^. As the membrane tension increases, the filament network with the narrow-angle pattern cannot support it in the movement direction. Consequently, the perpendicular actin filaments are rotated and bent under the increased load. Because the Arp2/3 complex prefers to bind on the convex side of bent filaments and generates daughter filaments with angles around 70° relative to the mother filaments^34^, this facilitates the configuration change of the network from the narrow-angle pattern into the ±35° pattern^8^ to meet the stiffness demand in the cell migration direction. However, when the load continues to increase, the stiffness of the filament network with the ±35° pattern is incapable of overcoming it, and the filaments are bent more severely. Thus, more Arp2/3 complex binds on the mother filaments and hence more daughter filaments grow out, which makes the network change from the ±35° pattern into the broad-angle pattern and have a higher stiffness to prevent the transverse and shear deformations in the migration plane. Thus, through the above adaptive behaviors, the remodeled and strengthened lamellipodial branched actin network can support the migrating cell to overcome the increased resistance. This resistance-adaptive intracellular biophysical mechanism (Fig. 8) interprets the experimental results^1,8^ that increased resistance load induces high lamellipodial actin network density.

In summary, as demonstrated in Fig. 8, our predictive assembling model reveals that migrating cells first can sensitively sense the variation of extracellular resistance through the bending deformations of actin filaments in the lamellipodial branched actin network. Then, based on the actin filament deformations, cells accordingly self-regulate the elastic properties of the branched actin network in a broad range through Arp2/3 nucleating, remodeling with F-actin, filamin-A, and α-actinin and altering actin filament orientations to adapt to the varying extracellular resistance. Such resistance-adaptive behaviors are versatile and essential in driving cell migration through highly varying and complex 3D confining extracellular microenvironments.

### Arp2/3 complex affects the stiffness of the branched actin network and cell migration from three aspects

Arp2/3 complex plays an essential role in regulating cell migration. We find that it can significantly influence the stiffness of the branched actin networks and then affect cell migration through three important mechanisms, i.e., successive branching generations, density, and distribution. The number of successive branching generations nucleated by it linearly enhances the stiffness of the branched actin network. Branched actin network with low successive branching generations is unable to support the driving force for cell migration. In addition, the increased branching density of the Arp2/3 complex significantly improves the network stiffness, which may explain why the Arp2/3 complex in metastatic cancer cells is dense^55,65^ and why cancer cells have strong migration abilities. Since a higher density of Arp2/3 complex means a stiffer lamellipodial branched actin network, it can enable cancer cells to overcome extracellular resistance more easily and thus to invade into other tissues and circulatory systems. However, when the F-actin concentration is limited, excessive high density of the Arp2/3 complex will inevitably result in severe local inhomogeneities of the lamellipodial branched actin network, and hence has an opposite effect: weakening the network stiffness. Nevertheless, cells can intelligently regulate the Arp2/3 complex branching density by some regulatory proteins, such as Profilin, Ena/VASP proteins, Arpin and Gadkin, to avoid extreme heterogeneity in the branched actin network^20,32,55^. Finally, our results also indicate that heterogeneity makes some local regions of the branched actin network stiffer, which may be related to the mechanisms of branched actin network steering cell migration.

### The unique elastic properties of the branched actin network are much different from those of the cross-linked actin network

Our results show that the Young’s and shear moduli of the lamellipodial branched actin network in the cell migration direction scale with the filament density to the power of 3.2 and 3.6 ($C_A^{3.2}$ and $C_A^{3.6}$, where *C*_*A*_ is the concentration of F-actin in the network), respectively, which significantly differ from the scaling power of 0.6 reported by the in vitro experiments^1^. Their experimental results showed that the scaling power of the branched actin network is much smaller than that ($C_A^{2.0}$) of the cross-linked actin network^44^. Conversely, our data demonstrate that compared to the cross-linked actin network, the stiffness of the branched actin network is much more sensitive to F-actin concentration. Since cross-linking proteins are much more flexible than actin filaments, the stiffness of the cross-linked actin network is mainly dominated by the weaker cross-linking proteins^57^. In the branched actin network, however, the branching junction nucleated by Arp2/3 complex is relatively rigid^9^. Thus, the stiffness of the branched actin network is more dominated by actin filaments rather than by cross-linking proteins (Supplementary Fig. 2). Such a stronger dependence on F-actin concentration of the branched actin network has important functional meaning in sensitive regulation of cell migrations through complex microenvironments. Our results have demonstrated that the uniformity of Arp2/3 complex branching is a key factor impacting the network stiffness. When we regulate the heterogeneity of the assembling model, the scaling power of the Young’s modulus in the cell migration direction is reduced from 3.2 to 2.2. For cellular materials, the Young’s modulus is proportional to the Young’s modulus of its constituent solid phase and proportional to the *k*th power of the solid relative density^66^. The exponent *k* varies for different types of cellular materials, and it is between 1.0 and 2.0 for open-cell foams^67^, and 1.0 and 3.0 for honeycombs (2D)^66^. When *k* approaches 1.0, the cellular material becomes a complete solid medium^66,67^. Thus, we speculate that the reasons for the very low scaling power 0.6 in the relevant experiments^1^ might be the extreme heterogeneity in the in vitro constructed branched actin network and the contribution from the experimental solution. In cells, however, there are some Arp2/3 complex regulatory proteins, which can tune the density of the Arp2/3 complex in the branched actin network and improve its efficiency in driving cell migration. More importantly, our study is based on predictive assembling models, which replicate both the microscopic and the macroscopic architectures of the lamellipodial sheet-like branched actin networks in migrating cells. Nevertheless, the published experiment is based on the in vitro constructed branched actin network^1^, whose structure might be very different from the in vivo sheet-like architecture.

Next, the stiffness of the branched actin network is several orders higher than that of the cross-linked actin network, which is only several Pa to several hundred Pa^49,57,68^. In addition, unlike the isotropic cross-linked actin network, the lamellipodial branched actin network is an orthotropic material, whose Young’s modulus in the cell migration direction and shear modulus in the cell migration plane are notably larger than other moduli. Such superior and special elastic properties have important consequences for ensuring the mechanical functions of supporting and steering cell migration. Importantly, finger-like filopodia, which provide another crucial way for migration cells mechanically sensing and splitting extracellular matrix, especially for tumor cell invasion and metastasis^2,69^, usually grow out from the lamellipodial branched actin network. When they protrude in confining extracellular matrix, they will produce high local load on the branched actin network. Therefore, the high elastic and shear moduli of the branched actin network also play an essential role in supporting the activities of filopodia and invadopodia. Finally, it should be noticed that this research focuses on studying how the intracellular proteins regulate the elastic properties of the branched actin network and then affect cell migration. Thus, the network is studied under small deformations and is simplified to be linear elastic. But the real structure shows viscoelastic behaviors under large deformations^1,26^.

### Why do lamellipodia persistently grow into sheet-like structures and directionally drive cell migration against resistances?

Under the resistance load from the cell migration direction, the bending of actin filaments is the dominant deformation mechanism of the branched actin network. Because the in-plane Poisson’s ratio is much larger than the out-of-plane one, when the cell migrates forward, actin filaments mainly undergo backward bending in the lamellipodial migration plane (*xy* plane in Fig. 2d) rather than in the out-of-plane (*yz* plane)). This feature is important for cell migration. Because the Arp2/3 complex prefers to bind on the convex side of a bent actin filament, the anisotropic feature of Poisson’s ratios promotes Arp2/3 complex nucleation and branching a daughter actin filament, which will have a small out-of-plane angle. Note that, the in-plane backward bending of actin filaments also essentially determines the polymerizing direction of the daughter filament to be in the direction of cell migration. This may explain why lamellipodia can persistently grow into sheet-like structures and grow toward the extracellular confining load. Thus, the dominant deformation mechanism and the effects of anisotropic Poisson’s ratios of the network and the Arp2/3 complex branching preference jointly determine why the lamellipodia grow into sheet-like structures and persistently protrude forward under extracellular confining resistance.

### Clinical values

Despite decades of experimental and clinical studies, cancer cell metastasis is still the major cause of mortality in patients^17–19^. To date, the underlying intracellular physical mechanism in regulating cell migration on a macromolecular level of proteins remain elusive^15,17,70^. Here, using the present predictive assembling model, we identify a resistance-adaptive intracellular mechanical self-regulation mechanism by which the lamellipodial branched actin network senses and adapts to varying extracellular resistances. Furthermore, this study systematically provides the quantitative relationships between the macroscopic elastic properties of the branched actin network and microscopic intracellular proteins, i.e., F-actin concentration, successive branching generations nucleated by Arp2/3 complexes, the density of Arp2/3 complex, and density of cross-linking proteins (filamin-A and α-actinin). In addition, the mechanical roles of the individual proteins in the process of lamellipodium supporting cell migration are clearly demonstrated. Therefore, these quantitative results have important clinical values and applications. For example, while clinical trials show that extracellular protease inhibitors, such as the matrix metalloproteinase inhibitor, have little effect as targets for anticancer therapy^71^, our results suggest that creating intracellular inhibitors for the Arp2/3 complex might be more effective for reducing cancer cell invasion and metastasis. Besides cancer metastasis, the physical mechanism revealed here also has important clinical values for the pathological problems of embryonic morphogenesis, wound healing, tissue renewal, and autoimmune disorders.

Furthermore, the Arp2/3 complex also participates in constructing other branched actin networks, which play central mechanical roles in endocytosis, phagocytosis, vesicle trafficking, intracellular pathogen transport, and dendritic spine formation^8,35^. Thus, the revealed elastic properties and mechanisms of the highly dynamic branched network also provide insights into the underlying physical mechanisms of endocytosis, phagocytosis, vesicle trafficking, intracellular pathogen transport, and dendritic spine formation.

## Methods

For the study of the micromechanical properties of cytoskeleton comprised of biopolymer network, FEM analysis based on a RVE model with periodic boundary conditions is an effective method^39,72–74^. The branched actin network in lamellipodium usually extends several micrometers from the leading edge to the rear^8,53,75^ and 20–50 µm along the leading edge^76^, and has a typical thickness of about 200 nm^64,77^. Therefore, it is suitable to construct RVE models in the migration plane and perform mechanical analysis using FEM.

However, during cell migration, the lamellipodial branched actin network is in a highly dynamic process interacting with various intracellular proteins and the fluctuating extracellular confining microenvironments. To construct continuum mechanics-based spatial periodic models for FEM mechanical analysis, we first need to simulate the dynamic and stochastic self-assembling process of the branched actin filament network in the sheet-like lamellipodial space and build its assembling mathematical model, which can realistically capture the self-assembling and remodeling behaviors of the branched actin network in driving cell migration. Then, this mathematical model is constructed into a self-assembling RVE model. In this process, 4600 lines of computer code are developed. By applying the experimentally measured geometric and elastic properties of the actin filament, Arp2/3 complex, filamin-A and α-actinin materials, and periodic boundary conditions to the RVE models, the effective elastic properties of the branched filament networks can be obtained by FEM analysis. Using this self-assembling RVE model, we can capture and study how the microscopic individual intracellular proteins and the extracellular confining resistance regulate the architecture of the branched actin network, respectively or jointly, and then regulate the macroscopic mechanical properties of the branched actin network for driving cell migration through varying extracellular microenvironment.

### Self-assembling mathematical model simulates the dynamic growth of the branched actin network driving cell migration

We develop computer codes to simulate the self-assembling and remodeling process of the 3D branched actin network in a sheet-like space by considering five types key proteins, namely, filamentous actin, Arp2/3 complex, capping protein, filamin-A and α-actinin, and their mechanochemical interactions, including actin polymerizing, Arp2/3 complex branching, capping protein-inhibiting polymerization, and cross-linking proteins’ binding and unbinding. All these intracellular proteins are assumed to be uniformly distributed in lamellipodia. This assumption is reasonable because they are coordinated by the treadmilling process between the actin polymerization and depolymerization^11,63^. In lamellipodia, the polymerization and depolymerization rates of actin are in a dynamic steady state^21^. Thus, here we only consider the net polymerization rate of actin filaments, which is given by^78^

1 $$V_g = \delta (k_{{\mathrm{on}}}M - k_{{\mathrm{off}}}),$$

where *δ* is the size of an actin monomer; *k*_on_ and *k*_off_ are the polymerization and depolymerization rates of actin filaments, respectively; and *M* is the molar concentration of actin monomers.

The relation between the total length of actin filaments and the concentration of filamentous actin (F-actin) in an RVE domain with the size of *w* × *w* × *h* is established as^79^

2 $$L = \frac{{C_AN_Aw^2hd_{{\mathrm{actin}}}}}{2},$$

where *L* is the total length of actin filaments; *C*_*A*_ is the concentration of F-actin; *w* and *h* are the in-plane side length of the selected lamellipodial RVE domain and the typical thickness of lamellipodia (200 nm), respectively; *N*_*A*_ is the Avogadro constant (6.02 × 10²³ mol^−1^), and *d*_actin_ is the diameter of actin monomers (~3.5 nm).

A lamellipodial RVE domain of 1000 × 1000 × 200 nm is selected to generate a certain number of pointed ends of mother actin filaments by referring to the concentration of F-actin based on Eq. (2). Note that since actin filaments are in a dynamic polymerization process during cell migration, the domain of the 1000 × 1000 nm square is only used to generate the pointed ends of mother filaments, but their polymerization is not confined in it. Specifically, both the $x_i^p$ and $y_i^p$ coordinates of the pointed end of the *i*th mother filament are randomly generated in the range from 0 to 1000. The $z_i^p$ coordinate of the pointed end is randomly generated by a Gaussian distribution function with a mean of 100 and a standard deviation of 50 because it is assumed that F-actin is more likely denser in the area nearing the central layer of a lamellipodium. Meanwhile, the value $z_i^p$ should be confined in the range of 0–200. To determine the orientation and the coordinates of the corresponding barbed end of every mother filament, a local spherical coordinate system is created by regarding every pointed end as the origin (Fig. 1d). In our simulation, the growth of actin filament by polymerization is completed in one step and is capped by a capping protein. The spherical coordinates $(r,\varphi ,\theta )$ of the barbed end are randomly generated by a normal or a uniform distribution as defined in Eq. (3). The polymerization length of the filaments, *r*, is generally in the range of 150–300 nm in lamellipodia^32,35,38^, and is determined by a random number from a normal distribution of *N*(250, 50). Because the length of actin filaments is normally larger than the thickness of lamellipodium, the polar angle $\varphi$ between actin filament and the positive *z* axis is confined in a narrow range and given by a random number from a uniform distribution of *U*(60°, 120°). Azimuthal angle $\theta$, the orientation of actin filaments relative to the cell moving direction in the *xy* plane, is commonly around ±35° for a cell with a medium moving velocity^61,62^ and is determined by a normal distribution of $N( \pm 35^\circ ,15^\circ )$. The shadow areas in Fig. 1d are the preferred range of the distribution of angle $\theta$. The coordinates of the barbed end $(x_i^b,y_i^b,z_i^b)$ for the *i*th mother actin filament in the 3D space are obtained by Eqs. (4) and (5).

3 $$r \sim N(250,50);\varphi \sim U(60^\circ ,120^\circ );\theta \sim N( \pm 35^\circ ,15^\circ \,^2),$$

4 $$\left( \begin{array}{l}x_i^b\\ y_i^b\\ z_i^b\end{array} \right) = r_i\left( \begin{array}{l}\sin \varphi \cos \theta \\ \sin \varphi \sin \theta \\ \cos \varphi \end{array} \right) + \left( \begin{array}{l}x_i^p\\ y_i^p\\ z_i^p\end{array} \right),$$

5 $$\left\{ {z_i^p|0 \le z_i^p \le 200} \right\}.$$

The diameter of actin filaments, *d*, is 7 nm^3^. After the generation of mother filaments, the Arp2/3 complex nucleates and binds on them randomly. To be consistent with experimental measurement, if there are two or more Arp2/3 complex binding on the same filament, there should be an interval *d*_*arp*_ between the two adjacent Arp2/3 branching points. *d*_*arp*_ is randomly generated from a uniform distribution of *U*(50, 150), which is a reasonable distance in lamellipodium^11,13^. The number of the Arp2/3 complex along an actin filament can be specified by the integer part of $r_i/d_{arp}$, where *r*_*i*_ is the length of the *i*th actin filament. Therefore, the coordinates of the *j*th starting point along the *i*th filament can be obtained as

6 $$\left( \begin{array}{l}x_{ij}^{as}\\ y_{ij}^{as}\\ z_{ij}^{as}\end{array} \right) = jd_{arp}\left( \begin{array}{l}\sin \varphi \cos \theta \\ \sin \varphi \sin \theta \\ \cos \varphi \end{array} \right) + \left( \begin{array}{l}x_i^p\\ y_i^p\\ z_i^p\end{array} \right).$$

The length $r_{ij}^{arp}$ and diameter of the Arp2/3 complex are ~10 nm^80^. It generates a branch from the mother filament by an angle of around 70°^9^. As a result, the possible branching position of Arp2/3 is constrained on a conical surface around the mother filament. In addition, the polar and azimuthal angles $\varphi _{ij}^{arp}$ and $\theta _{ij}^{arp}$ of the Arp2/3 complex in the spherical coordinate system should also satisfy the distributions defined by Eq. (3) to meet the relative orientation demand with respect to the direction of cell migration. Moreover, the filament length is normally larger than 100 nm in migrating lamellipodia^32,35,38^. Thus, the orientation of the Arp2/3 complex should also allow the forthcoming nucleated daughter filament to polymerize to a minimum length of 100 nm in the sheet-like lamellipodial space. If the coordinates of the *j*th ending point of the Arp2/3 complex are $(x_{ij}^{ae},y_{ij}^{ae},z_{ij}^{ae})$ in the global Cartesian coordinate system, the following constraint conditions must be satisfied:

7 $$\sqrt {(x_{ij}^{ae} - x_{ij}^{as})^2 + (y_{ij}^{ae} - y_{ij}^{as})^2 + (z_{ij}^{ae} - z_{ij}^{as})^2} = r_{ij}^{arp},$$

8 $$	\cos \alpha \\ 	\quad= \frac{{(x_i^b - x_i^p)(x_{ij}^{ae} - x_{ij}^{as}) + (y_i^b - y_i^p)(y_{ij}^{ae} - y_{ij}^{as}) + (z_i^b - z_i^p)(z_{ij}^{ae} - z_{ij}^{as})}}{{\sqrt {(x_i^b - x_i^p)^2 + (y_i^b - y_i^p)^2 + (z_i^b - z_i^p)^2} \sqrt {(x_{ij}^{ae} - x_{ij}^{as})^2 + (y_{ij}^{ae} - y_{ij}^{as})^2 + (z_{ij}^{ae} - z_{ij}^{as})^2} }},$$

9 $$\left\{ {\varphi _{ij}^{ae}|0 \le z_{ij}^{ae} + 100\cos \varphi _{ij}^{ae} \le 200} \right\},$$

where *α* is the angle between the mother filament and the Arp2/3 complex, and is randomly determined by a Gaussian distribution of *N* (70°^2^, 2°^2^). Based on the above constraint equations, the end-point coordinates $(x_{ij}^{ae},y_{ij}^{ae},z_{ij}^{ae})$ of the Arp2/3 complex are stochastically generated. After that, the daughter filaments begin to polymerize in the directions of the Arp2/3 complex from the same spherical coordinate system. Their growth lengths are also determined by the distribution given in Eq. (3). If actin filaments exceed the bottom (*z* = 0) or top (*z* = 200) surface of the lamellipodium in the *z* direction, they will be capped by capping proteins, and the polymerization will be stopped at the plane of *z* = 0 or *z* = 200. Using the same method, the next several generations of the Arp2/3 complex and daughter filaments are created from the already generated daughter filaments. Thus, the dendritic structure formed by actin filaments and Arp2/3 complex is constructed as shown in Fig. 2b, which is comparable to the inserted fluorescence microscopic image obtained in experiments^36^. The total length of actin filaments is determined by the concentration of F-actin and given by Eq. (2).

The cross-linking proteins (filamin-A and α-actinin) are produced to bind on the dendritic actin filaments, to connect them into an integrated branched actin network and to stabilize the lamellipodium. Instead of liking the cortex model in ref. ^39^, where cross-linking proteins are generated only according to the shortest distance between two filaments, and any two filaments can only be bound together by one cross-linking protein, we generate cross-linking proteins according to their connection properties (i.e., connection angle and distance) and the relative orientation and distance of the two filaments in the three-dimensional sheet-like space. Additionally, like the true condition in a migrating cell, two filaments can be cross-linked by several the same or different types of cross-linking proteins with the experimentally measured intervals. Filamin-A has a length of 160 nm and crosslinks two nearly orthogonal actin filaments (70–110°)^81^. The shortest cross-linking distance is ~30 nm^82^. Therefore, the cross-linking distance of filamin-A is in the range of 30–160 nm. In order to decide whether to generate filamin-A to crosslink two filaments, which are not connected by the same Arp2/3 complex, we first need to calculate the relative angle and the shortest distance between the two filaments. For example, for the *i*th filament with the pointed end of $(x_i^p,y_i^p,z_i^p)$ and barbed end of $(x_i^b,y_i^b,z_i^b)$ and the *j*th filament with a pointed end $(x_j^p,y_j^p,z_j^p)$ and barbed end $(x_j^b,y_j^b,z_j^b)$, their relative angle can be obtained by Eq. (10).

10 $$\beta = 	\frac{{180^\circ }}{\pi }\arccos \\ 	\quad\frac{{(x_i^b - x_i^p)(x_j^b - x_j^p) + (y_i^b - y_i^p)(y_j^b - y_j^p) + (z_i^b - z_i^p)(z_j^b - z_j^p)}}{{\sqrt {(x_i^b - x_i^p)^2 + (y_i^b - y_i^p)^2 + (z_i^b - z_i^p)^2} \sqrt {(x_j^b - x_j^p)^2 + (y_j^b - y_j^p)^2 + (z_j^b - z_j^p)^2} }},$$

11 $$\left\{ \beta |70^{\circ} \le \beta \le 110^{\circ} \cup 250^{\circ} \le \beta \le 290^{\circ} \right\}.$$

If they are appropriate for being cross-linked by filamin-A, the relative angle should satisfy Eq. (11). In addition, the shortest spatial distance between the two filaments $d_{\min }^{fls}$, which can be identified from Eqs. (12–16), should be in the range of cross-linking length of filamin-A as given by Eq. (17).

12 $$\left( \begin{array}{l}x_i\\ y_i\\ z_i\end{array} \right) = \left( \begin{array}{l}x_i^p\\ y_i^p\\ z_i^p\end{array} \right) + s\left( \begin{array}{l}x_i^b - x_i^p\\ y_i^b - y_i^p\\ z_i^b - z_i^p\end{array} \right),$$

13 $$\left( \begin{array}{l}x_j\\ y_j\\ z_j\end{array} \right) = \left( \begin{array}{l}x_j^p\\ y_j^p\\ z_j^p\end{array} \right) + t\left( \begin{array}{l}x_j^b - x_j^p\\ y_j^b - y_j^p\\ z_j^b - z_j^p\end{array} \right),$$

14 $$f(s,t) = (x_i - x_j)^2 + (y_i - y_j)^2 + (z_i - z_j)^2,$$

15 $$\left\{ \begin{array}{l}\frac{{\partial f(s,t)}}{{\partial s}} = 0\\ \frac{{\partial f(s,t)}}{{\partial t}} = 0\end{array} \right.$$

16 $$d_{\min }^{fls} = \left\{ \begin{array}{*{20}{c}} {\sqrt {(x_i - x_j)^2 + (y_i - y_j)^2 + (z_i - z_j)^2} } & {if\,{\mathrm{ }}0 {\,} \le {\,} s {\,} \le {\,} 1{\mathrm{ }}\,and\,{\mathrm{ }}0 {\,} \le {\,} t {\,} \le {\,} 1} \\ {\sqrt {(x_i - x_j^p)^2 + (y_i - y_j^p)^2 + (z_i - z_j^p)^2} } & {if\,{\mathrm{ }}0 {\,} < {\,} s {\,} < {\,} 1{\mathrm{ }}\,and\,{\mathrm{ }}t {\,} < {\,} 0} \\ {\sqrt {(x_i - x_j^b)^2 + (y_i - y_j^b)^2 + (z_i - z_j^b)^2} } & {if\,{\mathrm{ }}0 {\,} < {\,} s {\,} < {\,} 1{\mathrm{ }}\, and \,{\mathrm{ }}1 {\,} < {\,} t} \\ {\sqrt {(x_i^p - x_j)^2 + (x_i^p - x_j)^2 + (x_i^p - x_j)^2} } & {if\,s {\,} < {\,} 0\,and\,0 {\,} < {\,} t {\,} < {\,} 1} \\ {\sqrt {(x_i^b - x_j)^2 + (x_i^b - x_j)^2 + (x_i^b - x_j)^2} } & {if{\mathrm{ }}s {\,} < {\,} 0{\mathrm{ }}\,and\,{\mathrm{ }}0 {\,} < {\,} t {\,} < {\,} 1} \\ {\sqrt {(x_i^p - x_j^p)^2 + (y_i^p - y_j^p)^2 + (z_i^p - z_j^p)^2} } & {if\,{\mathrm{ }}s {\,} < {\,} 0{\mathrm{ }}\,and\,{\mathrm{ }}t {\,} < {\,} 0} \\ {\sqrt {(x_i^b - x_j^b)^2 + (y_i^b - y_j^b)^2 + (z_i^b - z_j^b)^2} } & {if\,{\mathrm{ }}1 {\,} < {\,} s{\mathrm{ }}\, and \,{\mathrm{ }}1 {\,} < {\,} t} \\ {\sqrt {(x_i^b - x_j^p)^2 + (y_i^b - y_j^p)^2 + (z_i^b - z_j^p)^2} } & {if\,{\mathrm{ }}1 {\,} < {\,} s{\mathrm{ }}\, and \,{\mathrm{ }}t {\,} < {\,} 0} \\ {\sqrt {(x_i^p - x_j^b)^2 + (y_i^p - y_j^b)^2 + (z_i^p - z_j^b)^2} } & {if\,{\mathrm{ }}s {\,} < {\,} 0{\mathrm{ }}\,and\,{\mathrm{ }}1 {\,} < {\,} t} \end{array} \right.$$

17 $$\left\{ d_{\min }^{fls}|30 {\,} \le {\,} d_{\min }^{fls} {\,} \le {\,} 160 \right\}.$$

On the basis of the above connection distance principles and relative orientation between two actin filaments, filamin-A is generated in the model. Experiments also showed that the shortest spacing between the two adjacent filamin-A binding on an actin filament is ~36 nm, which is the actin helical repeat^81^. Thus, to be consistent with the real condition in live cells, several filamin-A can be created along two filaments with the intervals of an integral multiple of the actin helical repeat in our mathematical model as long as they satisfy Eqs. (11) and (17). Another type of cross-linking protein existed in lamellipodia is α-actinin. Compared with filamin-A, α-actinin prefers to crosslink two paralleled actin filaments but also can crosslink two filaments with variable relative angles in lamellipodia^83,84^. Its linking distance is in the range from 24 nm to 40 nm^85,86^. The minimal interval between the adjacent α-actinin along an actin filament is ~31 nm^84,85^. Similar to filamin-A, α-actinin can be constructed to crosslink actin filaments according to its connection principles. In our mathematical model, both filamin-A and α-actinin are not permitted to crosslink the mother and daughter filaments connected by the same Arp2/3 complex. Additionally, it may be appropriate to generate both filamin-A and α-actinin in some locations in the model. It is assumed that filamin-A has the priority over α-actinin because the concentration of filamin-A in lamellipodium is higher than α-actinin^11^ and it has four binding sites while α-actinin has only 2, which enables filamin-A to have more opportunity to bind actin filaments. Although cross-linking protein reactions perform after the formations of dendritic actin structures by Arp2/3 in our assembling model, there is no difference in the final architectures between the branched actin networks generated asynchronously or synchronously. The finite element mechanical analyses are carried out after the final assembly of the network. Thus, they do not affect the elastic properties of the network. Because actin filaments are quite short (100–300 nm)^32,35,38^ and their density is small (3.0–10.0%) in lamellipodium^43^, there are few entanglements between them in the branched actin network. Moreover, compared with cross-linking proteins, entanglements are usually more fragile-like and easy to break^87^. Therefore, entanglements are deliberately ignored in our simulation.

To construct the self-assembling RVE model, we shift the parts of filaments, Arp2/3 complex, filamin-A, and α-actinin outside the square domain 1000 × 1000 nm (i.e., the RVE) into the domain by translating 1000 nm in the *x* or *y* direction (Fig. 2e) so that the RVE model is periodic in these directions. The diameters of actin filaments, Arp2/3 complex, and cross-linking proteins (filamin-A and α-actinin) are about 7 nm^3^, 10 nm^80^, and 4 nm^86^, respectively. They are also assigned to the RVE model. Thus, continuum mechanics-based hybrid biopolymer network models describing the dynamic lamellipodial branched actin networks are created. (Fig. 2d). Note that, both the microscopic and the macroscopic spatial reconfiguration of the network, which is induced by the varying extracellular confining resistance in cell migration process, can be realistically simulated by this RVE model through regulating the Arp2/3 complex nucleation, F-actin, filamin-A, α-actinin self-assembling and disassembling, and actin filament-polymerizing orientations.

### Validation with published experimental data

As shown in Fig. 2b, the architecture generated in our RVE model is very similar to the experimental images of the branched actin network in ref. ^13^ and ref. ^36^. In addition, as expected, it can be seen from Fig. 2c that with the increase of filament density *V*_*f*_, both the numbers of filamin-A and α-actinin increase with growing gradients because higher *V*_*f*_ means more appropriate cross-linking positions between filaments. For the usual density range (3.0–10.0%) of actin filaments^43^, the number of the Arp2/3 complex in the RVE model is larger than that of filamin-A, which, in turn, is larger than that of α-actinin. This is consistent with the experimental measurements of the relative densities of connection proteins^11^: the density of the Arp2/3 complex is larger than that of filamin-A, and the latter, in turn, is larger than that of actinin in the branched actin network in lamellipodium. Because the average interval *darp* between two adjacent Arp2/3 complexes along an actin filament in models is based on experimental measurements^11,13^, the number of Arp2/3 in the RVE model is a control parameter and reflects its realistic density in lamellipodia. Therefore, our model can successfully predict the densities of filamin-A and α-actinin, which are fitting parameters in the model. Moreover, as shown in Supplementary Table 4 (Supplementary Information), our RVE models with an actin filament density of 7.8% have ~290 filaments per micron at the cross-section of *y* = 1000, which agrees well with the experimental data^43^ that there are ~300 filaments per micron length of lamellipodium margin in keratinocyte and fibroblast, whose actin filament density of the branched actin network in lamellipodia is also normally 7.8%^79^.

### Mesh and boundary conditions

The hybrid branched and cross-linked actin filament network in the RVE model (Fig. 2b) has meshed into quadratic interpolated B32 beam elements with circular cross-sections in ABAQUS simulations. This element type is based on the Timoshenko beam theory allowing for bending, torsion, axial compression/stretching, and transverse shear deformations. The solid materials of actin filaments and cross-linking proteins are assumed to be isotropic and linearly elastic, whose Young’s moduli and Poisson’s ratios are obtained from the literature and given in Supplementary Table S1. According to experimental measurements, filamin-A and α-actinin have similar mechanical performances^59^, and thus are assumed to have the same mechanical properties. Compared with actin filaments and crosslinkers, the dimensions of the Arp2/3 complex are very small (assumed to be a cylinder with both diameter and length being 10 nm)^80^ and the connections formed by it between the mother and daughter actin filaments are relatively rigid^9^. Thus, the elastic properties of the Arp2/3 complex are assumed to be the same as those of actin filaments. The diameters and elastic properties of actin filaments and cross-linking proteins are obtained from refs. ^58,60,88,89^, as shown in Supplementary Table S1.

Periodic boundary conditions are applied to the RVE models (Fig. 2b) in the *x* and *y* directions. Constraint equations of the periodic boundary nodes for meeting the continuity and equilibrium of adjacent RVEs are given by Eqs. (18)–(25).

18 $$u_i^{x = 0} - u_{i\prime }^{x = 1000} = u_j^{x = 0} - u_{j\prime }^{x = 1000},$$

19 $$v_i^{x = 0} - v_{i\prime }^{x = 1000} = v_j^{x = 0} - v_{j\prime }^{x = 1000},$$

20 $$w_i^{x = 0} - w_{i\prime }^{x = 1000} = w_j^{x = 0} - w_{j\prime }^{x = 1000},$$

21 $$\theta _i^{x = 0} = \theta _{i\prime }^{x = 1000},$$

22 $$u_i^{y = 0} - u_{i\prime }^{y = 1000} = u_j^{y = 0} - u_{j\prime }^{y = 1000},$$

23 $$v_i^{y = 0} - v_{i\prime }^{y = 1000} = v_j^{y = 0} - v_{j\prime }^{y = 1000},$$

24 $$w_i^{y = 0} - w_{i\prime }^{y = 1000} = w_j^{y = 0} - w_{j\prime }^{y = 1000},$$

25 $$\theta _i^{y = 0} = \theta _{i\prime }^{y = 1000},$$

where *u*, *v*, and *w* denote the displacements in the *x*, *y*, and *z* directions, respectively. *i* and *j* are the nodes on the boundary of *x* = 0 or *y* = 0, while $i\prime$ and $j\prime$ are their corresponding nodes on the opposite boundary (i.e., *x* = 1000 or *y* = 1000), respectively. $\theta$ represents the rotational angles around the *x*, *y*, and *z* axes.

On the top and bottom surfaces of a lamellipodium, the branched actin filament network is constrained by the membrane. Therefore, all nodes on the boundary of *z* = 0 are assumed to have zero displacement in the *z* direction, and all nodes on the boundary of *z* = 200 are assumed to have the same displacement in the z direction, which can be determined by Eq. (28).

26 $$w_i^{z = 0} = 0,$$

27 $$w_{i\prime }^{z = 200} = w_{j\prime }^{z = 200},$$

28 $$\mathop {\sum}\limits_{i\prime = 1}^n {F_{zi\prime }^{z = 200} = 0},$$

where *w* denotes the displacement in the *z* direction; *i* are the nodes on the $z = 0$ boundary; $i\prime$ and $j\prime$ are the nodes on the $z = 200$ boundary; $F_{zi\prime }$ and *n* are the force component in the *z* direction of node $i\prime$ and the total number of nodes on the $z = 200$ boundary, respectively.

### Elastic constants

Under the imposed boundary displacements, the total energy of the RVE model is the sum of the strain energies of bending, axial, shear, and torsion deformations of actin filaments, Arp2/3 complex, filamin-A, and α-actinin, and can be expressed as

29 $$U_{{\mathrm{total}}} 	= \frac{1}{2}\mathop{\sum}\limits_{ < ij > } \int \left(E_{s} I\left(\frac{{d\theta (s_{ij})}}{{ds_{ij}}}\right)^{2}+ E_{s} A\left(\frac{{du(s_{ij})}}{{ds_{ij}}}\right)^{2} + \lambda G_{s} A\left(\frac{{dv(s_{ij})}}{{ds_{ij}}} - \theta (s_{ij})\right)^{2} \right.\\ 	 \quad + \left. G_{s} J\left(\frac{{d\varphi (s_{ij})}}{{ds_{ij}}}\right)^{2}\right)ds_{ij},$$

30 $$G_s = \frac{{E_s}}{{2(1 + v_s)}},$$

where *i*, *j*, and *s*_*ij*_ are, respectively, the two vertices and length of a segment of actin filaments, Arp2/3 complex, filamin-A, or actinin in the RVE model; *E*_*s*_ and *G*_*s*_ are their Young’s and shear moduli; *A*, *I*, and *J* are the area, the second moment, and polar second moment of their cross-sections, respectively. $u(s_{ij})$ and $v(s_{ij})$ are axial and transverse displacements. $\theta (s_{ij})$ and $\varphi (s_{ij})$ are the rotation and torsion angles of the centroidal axis of the fiber segment; and $\lambda = 10/9$ is the transverse shear coefficient of the circular cross-section. Based on the minimum total potential energy principle (Eq. (31)), the system equilibrium deformation state can be solved.

31 $$\prod _p^ \ast \ge \prod _p,$$

where $\prod _p$ and $\prod _p^ \ast$ are the true and possible total energies of the system, respectively. The effective elastic modulus of the bulk network can be calculated by

32 $$E_k = \frac{{\mathop {\sum}\limits_i {(f_k)_iw} }}{{(wh)d_k}} = \frac{{\mathop {\sum}\limits_i {(f_k)_i} }}{{hd_k}},$$

where *d*_*k*_ is the imposed displacement in direction *k*. *w* and *h* are the side length and thickness of the RVE model (Fig. 2b). (*f*_*k*_)_*i*_ is the reaction force in direction *k* of the *i*th node on the boundary whose normal direction is *k*. As can be seen from Supplementary Tables 2 and 3 (Supplementary Information), the elastic constants of the branched actin network obtained from uniaxial compression or tension tests satisfy the following relation

33 $$\frac{{v_{ij}}}{{E_j}} = \frac{{v_{ji}}}{{E_i}}{\mathrm{(}}i,j = 1,2,3\,and\,i \, \ne \, j{\mathrm{)}},$$

where Poisson’s ratios are defined as $v_{ij} = - \varepsilon _i/\varepsilon _j$, and $\varepsilon _i$ is the normal strain in direction *i* when uniaxial stress is applied in the direction *j*; *E*_*i*_ is the Young’s modulus in the *i* direction; 1, 2, and 3 represent the *x*, *y*, and *z* directions, respectively. The branched actin filament network material has three orthogonal planes of elastic symmetry. Thus, normal stresses only generate normal strains, and each shear stress only generates the corresponding shear strain independently; and in order to fully describe the elastic mechanical behaviors of this model, nine independent elastic constants (*E*_1_, *E*_2_, *E*_3_, *G*_12_, *G*_23_, *G*_31_, *v*_12_, *v*_23_, *v*_31_) are required because the compliance matrix is symmetric, and the RVE model has three orthogonal planes of elastic symmetry. *G*_*ij*_ is the shear modulus in the *ij* plane. The constitutive relationship of the branched actin filament network material is given by

34 $$\left( \begin{array}{l}\varepsilon _{11}\\ \varepsilon _{22}\\ \varepsilon _{33}\\ \gamma _{12}\\ \gamma _{23}\\ \gamma _{31}\end{array} \right) = \left( {\begin{array}{*{20}{c}} {\frac{1}{{E_1}}} & { - \frac{{v_{12}}}{{E_2}}} & { - \frac{{v_{13}}}{{E_3}}} & 0 & 0 & 0 \\ { - \frac{{v_{21}}}{{E_1}}} & {\frac{1}{{E_2}}} & { - \frac{{v_{23}}}{{E_3}}} & 0 & 0 & 0 \\ { - \frac{{v_{31}}}{{E_1}}} & { - \frac{{v_{32}}}{{E_2}}} & {\frac{1}{{E_3}}} & 0 & 0 & 0 \\ 0 & 0 & 0 & {\frac{1}{{G_{12}}}} & 0 & 0 \\ 0 & 0 & 0 & 0 & {\frac{1}{{G_{23}}}} & 0 \\ 0 & 0 & 0 & 0 & 0 & {\frac{1}{{G_{31}}}} \end{array}} \right)\left( \begin{array}{l}\sigma _{11}\\ \sigma _{22}\\ \sigma _{33}\\ \sigma _{12}\\ \sigma _{23}\\ \sigma _{31}\end{array} \right).$$

### Statistics and reproducibility

Each data point is an average value calculated from about 30 stochastic models, which have the same intracellular protein parameters. The error bar is the standard deviation of the results from these models. All these results in this study can be reproduced by constructing these models and then by applying commercial software ABAQUS2017 to perform finite element simulations.

## Supplementary information

Supplementary Information

## Data Availability

All the relevant data that support the findings of this study are available from the corresponding author on reasonable request.

## Code availability

Computer code used to simulate the stochastic dynamic growth of the branched actin network in lamellipodia, construct the RVE model, and perform finite element analysis are available from the corresponding author on reasonable request. The finite element software used in this study is ABAQUS2017.
