# Author Correction: Predictive assembling model reveals the self-adaptive elastic properties of lamellipodial actin networks for cell migration

**DOI:** 10.1038/s42003-021-02180-4

**Published:** 2021-05-13

**Authors:** Xindong Chen, Hanxing Zhu, XiQiao Feng, Xiaona Li, Yongtao Lu, Zuobin Wang, Yacine Rezgui

**Affiliations:** 1 School of Engineering, Cardiff University, Cardiff, CF24 3AA UK; 2 Institute of Biomechanics and Medical Engineering, AML, Department of Engineering Mechanics, Tsinghua University, 100084 Beijing, China; 3 School of Computer Science, Northwestern Polytechnical University, 710129 Xi’an, China; 4 State Key Laboratory of Structural Analysis for Industrial Equipment, Dalian University of Technology, 116024 Dalian, China; 5 International Research Centre for Nano Technology of China, Changchun University of Science and Technology, 130022 Changchun, China

**Keywords:** Biophysics, Motility, Cellular motility

Correction to: *Communications Biology* 10.1038/s42003-020-01335-z, published online 26 October 2020.

In the original version of the Article, Figure 3 panels b–d displayed incorrect units for F-actin Concentration, C_A_. The units were given as mA. The correct units are mM. The error has been corrected in the HTML and PDF versions of the Article.

