# Supplementary material for: Predictive assembling model reveals the self-adaptive elastic properties of lamellipodial actin networks for cell migration
Source: Commun Biol. 2020 Oct 26;3:616. doi: 10.1038/s42003-020-01335-z (PMC7588425; doi:10.1038/s42003-020-01335-z)
Supplement: Supplementary file 1 — Supplementary Information [file 42003_2020_1335_MOESM1_ESM.pdf]

Supplementary Information for

Predictive assembling model reveals the self-adaptive elastic properties of lamellipodial actin network for cell migration

Xindong Chen[1], Hanxing Zhu[1*], XiQiao Feng[2], Xiaona Li[3], Yongtao Lu[4], Zuobin Wang[5], Yacine Rezgui[1]

[1] School of Engineering, Cardiff University, Cardiff CF24 3AA, UK

[2] Institute of Biomechanics and Medical Engineering, AML, Department of Engineering Mechanics, Tsinghua University, Beijing 100084, China

[3] School of Computer Science, Northwestern Polytechnical University, Xi'an 710129, China

[4] State Key Laboratory of Structural Analysis for Industrial Equipment, Dalian University of Technology, Dalian, China

[5] International Research Centre for Nano Handling and Manufacturing of China, Changchun University of Science and Technology, Changchun 130022, China

This PDF file includes:

Supplementary information text

Supplementary Tables 1 to 5

SI References

Supplementary Information Text

Supplementary Information of Intracellular Proteins

Filament density (F-actin concentration). According to electron microscopy investigation, the concentration of F-actin in lamellipodia is normally in the range of 0.5 mM to 2 mM [1, 2] and can reach up to 10 mM in some local areas [3, 4]. Using Eq. (S1) in the main text, the total length of actin filaments can be calculated, which agrees well with the experimental measurements, i.e., 180~500 μm total length of filaments per μm^2 in the lamellipodia [5]. Based on the dimensions of actin filaments and lamellipodia, the actin filament density of the branched actin filament networks in lamellipodia can be obtained in the range of 3%~10%. In the RVE model, the density (or solid vlume fraction) of actin filaments can be derived as

$$V_f = \frac{\sum_{i=1}^{N^{filament}} r_i \pi d^2}{4w^2 h} \tag{S1}$$

where $N^{filament}$ is the total number of actin filaments in the RVE model; r_i is the length of the *i*th actin filament; d is the diameter of actin filaments; w and h are the side length and thickness of the RVE model (Main text, Fig. 2d), respectively.

Number of successive branching generations formed by Arp2/3 complex. We have studied the effect of the number K of successive branching generations from a mother filament and explored its possible value on the basis of the protrusion force generated per filament. In order to exclude the impact of Arp2/3 complex density, the average interval d_{arp} along actin filaments for forming one Arp2/3 complex is fixed by using the same

uniform distribution ($U(50,150)$) in all models. Here we take the lamellipodia of keratocytes as example in our main text, whose density of actin filaments in the branched actin network is about 7.8%. When a cell moves or spreads, using the polymerization force generated by actin filaments and the Young's modulus E_2 of the branched actin network obtained from our finite element simulations, the compressive stress σ and strain ε of the branched actin network in the migration direction (i.e. the *y* direction) can be obtained as

$$\sigma = \frac{nf_p}{wh} \tag{S2}$$

$$\varepsilon = \frac{\sigma}{E_2} \tag{S3}$$

where n is the total number of actin filaments that push against the leading edge membrane of lamellipodium; f_p is the average polymerization force (in the cell movement direction) generated by an actin filament.

Density of Arp2/3 complex. Experiments showed that the concentration of Arp 2/3 complex plays an essential role in cell motility by affecting the branching density in the branched actin network [6, 7]. We examined the effects of branching density on the elastic properties of branched actin network while the subnetworks were kept having 3 successive branching generations. Because the branching points formed by Arp2/3 complex are relatively rigid, the average value of d_{arp}, which is defined as the average distance of two adjacent Arp2/3 complex along a filament, is also called the characteristic length l_c in the branched actin filament [8]. The average density of Arp 2/3 complex is defined as n_{arp}, which

is the average number of Arp 2/3 complex along the average length of actin filaments r_a.

Parameter n_{arp} can be obtained from the following equation

$$N_m(n_{arp}^3 + n_{arp}^2 + n_{arp}) = N_{arp} \tag{S4}$$

where N_m and N_{arp} are the total number of the mother filaments and the total number of Arp 2/3 complex in the model, respectively.

To quantify the network heterogeneity, the RVE model is equally divided into κ parts in the *xy* plane and the density of actin filaments in each part is calculated. Then, the network heterogeneity Ω can be defined as

$$\Omega = \frac{1}{V_f}\sqrt{\frac{1}{\kappa}\sum_{i=1}^{\kappa}(V_{fi} - V_f)^2} \tag{S5}$$

where V_{fi} is the filament density of the *i*th part. V_f is the actin filament density of the RVE model and it is also the mean filament density of all the κ parts.

Densities of flexible crosslinking proteins. Crosslinking proteins crosslink and stabilize the dendritic subnetworks into an integrated branched actin network [9-15]. Theoretical analysis shows that crosslinking connectivity between fibres is a key parameter influencing the mechanical behavior of fibre networks [16]. Experiments show that mutations and dysfunction of crosslinking proteins significantly impact the mechanical performance of cytoskeleton [17, 18]. The density of crosslinking proteins (including both filamin-A and α-actinin) ρ_a is defined as the average number of them along the average length of a filament, and given by

$$\rho_c = r_a N_c / L \tag{S6}$$

where N_c is the total number of filamin-A and α-actinin, r_a is the average length of actin filaments and L is the total length of actin filaments in the RVE model (Main text, Fig. 1g). The individual density of filamin-A ρ_f or α-actinin ρ_a can also be obtained by using Eq. (S6) by replacing N_c by the total number of filamin-A or α-actinin, respectively. To quantitatively assess the impact of crosslinking density on the elastic properties of the branched actin network, we develop the models by giving different possibilities p_c of 1.0, 0.9, 0.8, 0.7, 0.6, 0.5, 0.4 and 0.3 for generating a cross-linker between the two possible points of two filaments to simulate the assembling or disassembling of crosslinking proteins during cell migration.

Supplementary Figure 1 Schematic operation of generating actin filaments, Arp2/3 complex and crosslinking proteins (filamin-A and α-actinin) on the boundaries of an in-plane periodic RVE model.

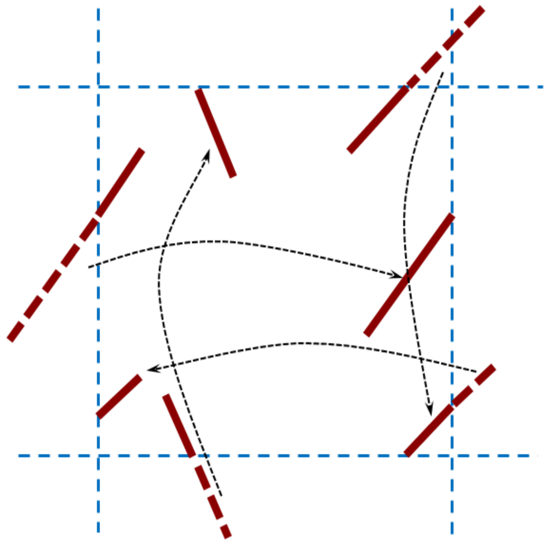

Supplementary Figure 2 The dimensionless Young's and shear moduli of the branched actin network when the Young's modulus of actin filaments is $10E_f$ or $0.1E_f$ and the Young's modulus of crosslinking proteins is $10E_c$ or $0.1E_c$, respectively. The results are normalized by the Young's or shear modulus of the branched actin network when the Young's moduli of actin filaments and crosslinking proteins are E_f and E_c.

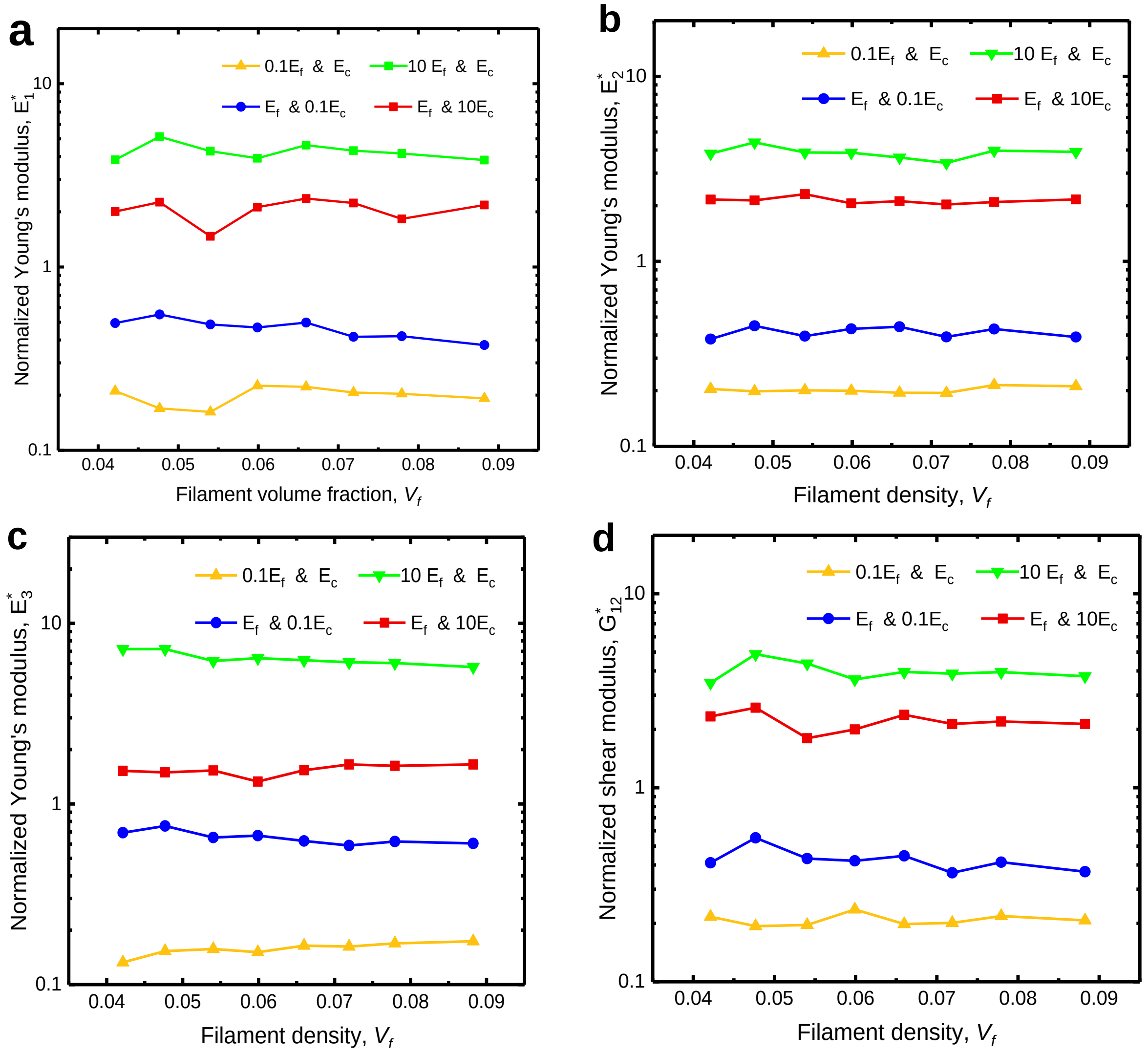

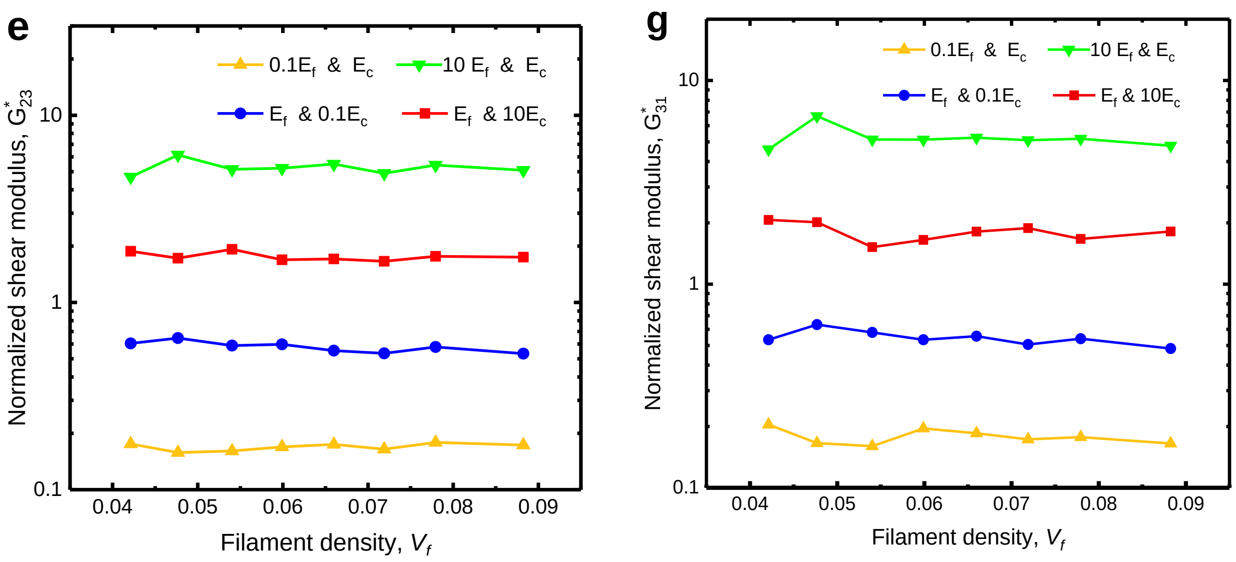

a Young's modulus E_1^*. **b** Young's modulus E_2^*. **c** Young's modulus E_3^*. **d** Shear modulus G_{12}^*. **e** Shear modulus G_{23}^*. **f** Shear modulus G_{31}^*.

Supplementary Figure 3 Statistic results of crosslinking proteins and elastic moduli obtained from more than 2400 stochastic models. Each data point is a mean value calculated from 30 models.

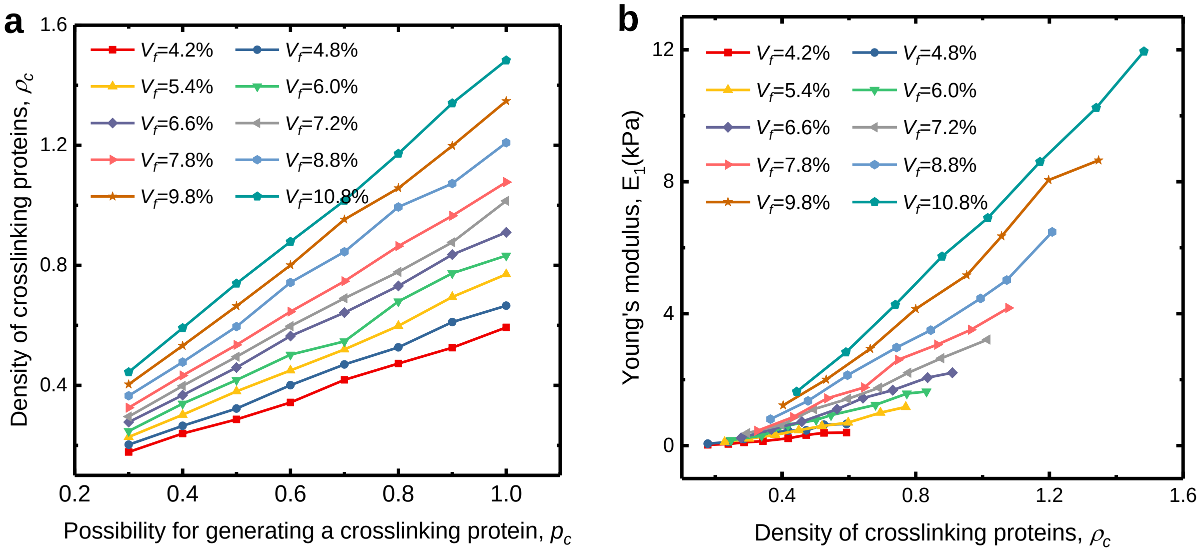

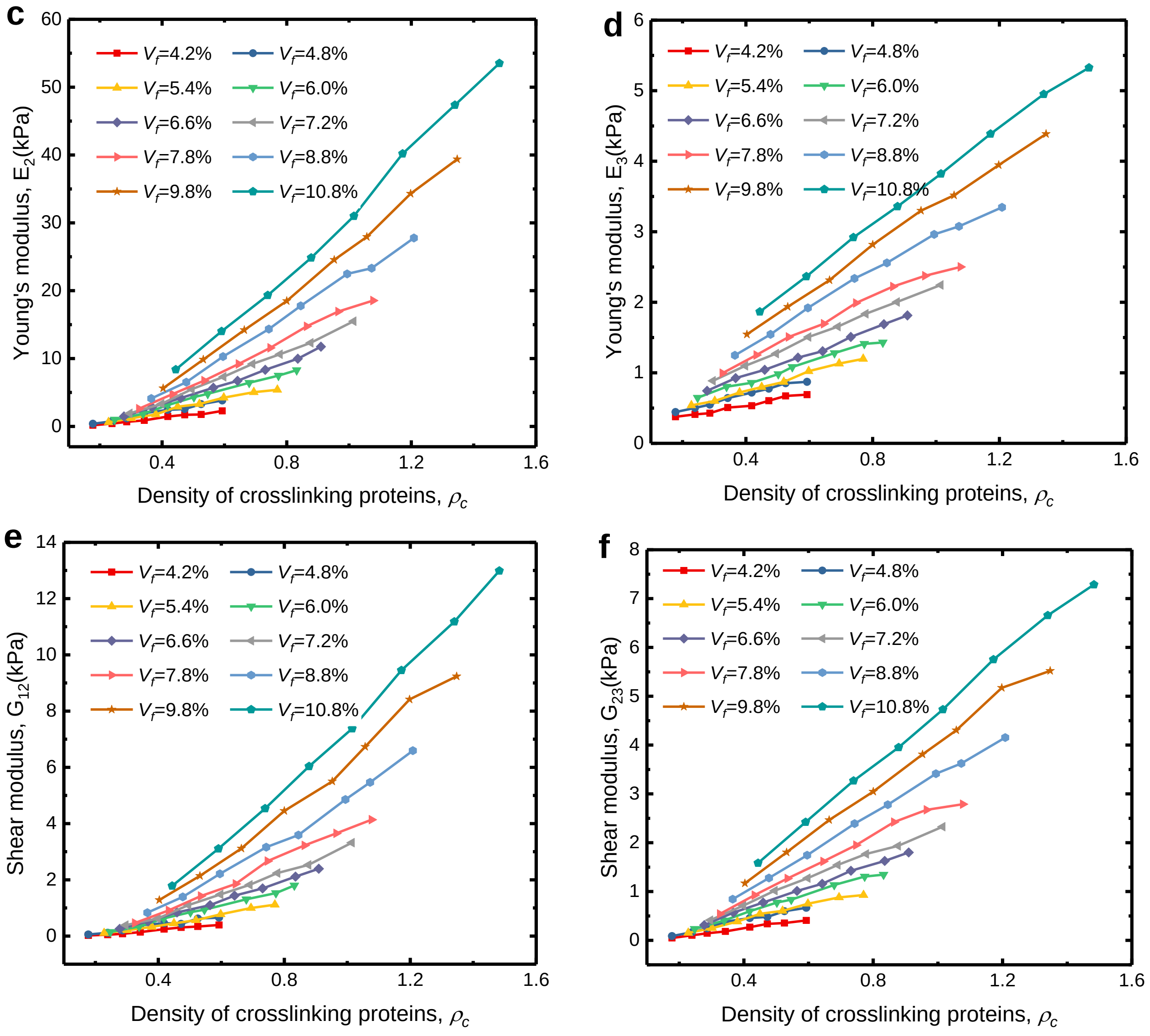
c
Young's modulus, E_2(kPa)
Density of crosslinking proteins, ρ_c
d
Young's modulus, E_3(kPa)
Density of crosslinking proteins, ρ_c
e
Shear modulus, G_{12}(kPa)
Density of crosslinking proteins, ρ_c
f
Shear modulus, G_{23}(kPa)
Density of crosslinking proteins, ρ_c
V_f=4.2%
V_f=4.8%
V_f=5.4%
V_f=6.0%
V_f=6.6%
V_f=7.2%
V_f=7.8%
V_f=8.8%
V_f=9.8%
V_f=10.8%

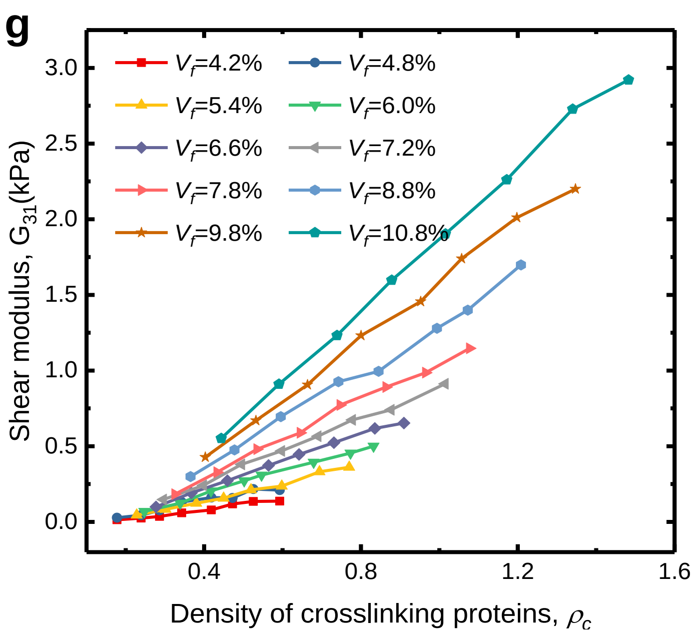

a The density of crosslinking proteins (filamin-A and α-actinin) under different combinations of filament density V_f and generation possibility p_c. **b-g** Elastic properties under different combinations of filament density V_f and the density of crosslinking proteins ρ_c. **b** Young's modulus E_1. **c** Young's modulus E_2. **d** Young's modulus E_3. **e** Shear modulus G_{12}. **f** Shear modulus G_{23}. **g** Shear modulus G_{31}.

Supplementary Table 1 Diameters and elastic properties of actin filaments and crosslinking proteins

Types of proteins	Diameter of cross-section	Poisson's ratio	Young's modulus	Refs.
Actin filaments	7 nm	0.3	2 Gpa	[19-21]
Crosslinking proteins (filamin-A and α-actinin)	4 nm	0.3	60 Mpa	[22]

Supplementary Table 2 Elastic properties obtained from uniaxial tension, compression and pure shear tests at filament density of 7.8%.

NO.	V_f	E_1	v_{21}	v_{31}	E_2	v_{12}	v_{32}	E_3	v_{13}	v_{23}	G_{12}	G_{23}	G_{31}
1	0.0778522	4.2678778	0.0940823	0.0107896	20.614739	0.454437	0.1036847	3.1668505	0.0080062	0.0159281	3.8039541	3.3630058	1.2642774
2	0.0792666	5.0243766	0.0962565	0.0193171	17.63162	0.3377849	0.0496505	2.8521148	0.0109654	0.0080315	4.2306291	2.6399285	1.1847965
3	0.0771606	3.7419244	0.1207253	0.0701783	13.85511	0.4470061	0.1775703	2.017227	0.0378323	0.0258532	3.4785341	2.0618086	0.9480159
4	0.0780412	5.5472525	0.1151662	0.0557327	16.702145	0.3467524	0.1003146	2.3234713	0.0233436	0.013955	3.9214906	2.635923	1.3032033
5	0.0768723	2.9230772	0.0857754	0.0765217	14.16518	0.4156666	0.1461422	2.0510438	0.0536932	0.0211606	3.2755838	2.4807765	1.0088077
6	0.0771209	3.9816797	0.0803671	0.0623932	17.9945	0.3632048	0.1969901	2.4995533	0.0391681	0.0273632	3.2167591	2.7780695	1.1669275
7	0.0785634	4.2849766	0.0796984	0.0359364	20.172541	0.3751989	0.2095834	2.486744	0.0208553	0.0258361	4.6956616	3.1840253	1.145986
8	0.0787042	4.8105972	0.1160545	0.0158504	25.545948	0.61629	0.3894709	2.4860765	0.0081913	0.0379025	6.1795781	3.1765928	1.4013368
9	0.0766642	3.5332456	0.0784434	-0.0012	18.989353	0.4215921	0.155589	2.0047291	-0.000681	0.0164257	2.7375534	2.5943198	0.8740904
10	0.078482	4.5412194	0.0597446	0.0480986	15.679609	0.2062821	0.2550507	2.110767	0.0223565	0.0343398	3.2832644	2.466487	1.0488424
11	0.0782078	4.1816981	0.0890421	0.069263	20.511339	0.436754	0.0533849	2.1503483	0.035617	0.0055967	4.8359572	3.1812485	1.1190167
12	0.0786592	4.6561181	0.1328038	0.0599033	23.108978	0.6591241	0.0605109	3.0369098	0.0390714	0.0079521	5.6760994	3.255354	1.4256858
13	0.0792858	4.3561747	0.1034888	0.0342232	23.564185	0.5598098	0.243775	2.7082838	0.021277	0.0280176	5.4312688	3.6277938	1.3157345
14	0.0765458	4.7955925	0.1442149	0.0586948	17.184436	0.5167769	0.2419162	2.682194	0.0328283	0.0377589	4.6900256	2.909648	1.2631975
15	0.077912	4.8739016	0.0652526	0.05947	19.601748	0.3955753	0.1671987	2.463021	0.0303461	0.0163618	4.3468909	2.6012198	1.2359605
16	0.0779638	3.9286531	0.1066913	0.0424206	19.304405	0.4785685	0.1863177	2.592608	0.0265858	0.023443	3.9924441	3.0921485	1.150878
17	0.0788466	3.8822397	0.1166994	0.0256228	16.775916	0.4644003	0.1779509	2.4915293	0.0177641	0.0212088	4.0073034	2.6035683	1.007417
18	0.0780435	4.0378125	0.1228836	0.0368926	18.167973	0.5529096	0.2442945	1.9488515	0.0178062	0.0262051	4.3874763	2.3978463	1.0167932
19	0.0770202	4.2241363	0.119251	0.0049068	14.608055	0.4123979	0.1730022	2.3752303	0.0027591	0.0281297	3.669225	2.3978763	0.9771235
20	0.0770612	3.5938134	0.112496	0.0279667	18.707518	0.5855956	0.1996629	3.005836	0.0233912	0.0320809	4.0125828	2.9531295	1.1459111
21	0.0765107	4.0981138	0.1194188	0.0802675	20.492428	0.5971482	0.3156111	2.3124925	0.0452935	0.0356155	4.5749059	3.1770935	1.2366593
22	0.0797588	5.642365	0.175075	0.0789544	17.821861	0.5529885	0.1794574	2.6204168	0.0366679	0.0263863	4.5555672	2.777565	1.3265745
23	0.0774463	2.2097366	0.0864393	0.0313045	14.563214	0.5696757	0.1882368	2.1272375	0.0301357	0.0274956	2.7335994	2.5983623	0.6794959
24	0.0778448	4.6102844	0.14059	0.0325993	18.238496	0.5561805	0.1260762	2.648472	0.0187273	0.0183079	5.5051769	2.4909135	1.1921749
25	0.0771923	4.4607059	0.09891	0.0170179	17.60364	0.3903363	0.1160297	2.6349368	0.0100525	0.0173675	3.9358297	2.5376955	1.1978278
26	0.0768698	3.43743	0.0668624	-0.034843	18.568499	0.3611813	0.0935916	2.9745265	-0.030151	0.0149926	3.4933303	2.8012683	1.1250177
27	0.0786199	2.9962525	0.0621988	0.0132914	21.413665	0.4445231	0.163905	2.791442	0.0123829	0.0213663	3.2703084	2.7799155	0.8966991
28	0.0778698	4.0795019	0.1364903	0.0122197	16.09174	0.5383911	0.1762109	2.1576678	0.006463	0.0236273	4.0499259	2.2417545	1.0299721
29	0.0795129	4.3093334	0.0811484	0.0632936	22.014955	0.4145603	0.1662105	2.563016	0.0376445	0.0193505	4.0896959	3.02968	1.1907323
30	0.0790069	4.2832084	0.1043167	0.0556636	16.77212	0.4084816	0.260241	2.7940193	0.0363108	0.0433527	4.0249734	2.7485953	1.4839206
Average	0.0779635	4.17711	0.1036862	0.0387584	18.548864	0.4626531	0.1772543	2.5025872	0.0224901	0.0233804	4.1368532	2.7861204	1.1454359
Std.	0.0009176	0.7135686	0.0267501	0.0270037	2.7861428	0.0992592	0.0739765	0.3291576	0.0164743	0.0090594	0.8167818	0.3525769	0.1728945

Supplementary Table 3 Relationships between the elastic constants of the branched actin filament network with a filament density of 7.8%.

NO.	V_f	v_{21}/E_1	v_{12}/E_2	v_{31}/E_1	v_{13}/E_3	v_{32}/E_2	v_{23}/E_3
1	0.0778522	0.0220443	0.0220443	0.0025281	0.0025281	0.0050296	0.0050296
2	0.0792666	0.0191579	0.0191579	0.0038447	0.0038447	0.002816	0.002816
3	0.0771606	0.0322629	0.0322629	0.0187546	0.0187546	0.0128162	0.0128162
4	0.0780412	0.020761	0.020761	0.0100469	0.0100469	0.0060061	0.0060061
5	0.0768723	0.0293442	0.0293443	0.0261785	0.0261785	0.010317	0.010317
6	0.0771209	0.0201842	0.0201842	0.0156701	0.0156701	0.0109472	0.0109472
7	0.0785634	0.0185995	0.0185995	0.0083866	0.0083866	0.0103895	0.0103895
8	0.0787042	0.0241248	0.0241248	0.0032949	0.0032949	0.0152459	0.0152459
9	0.0766642	0.0222015	0.0222015	-0.00034	-0.00034	0.0081935	0.0081935
10	0.078482	0.0131561	0.0131561	0.0105916	0.0105916	0.0162664	0.0162689
11	0.0782078	0.0212933	0.0212933	0.0165634	0.0165634	0.0026027	0.0026027
12	0.0786592	0.0285224	0.0285224	0.0128655	0.0128655	0.0026185	0.0026185
13	0.0792858	0.0237568	0.0237568	0.0078563	0.0078563	0.0103451	0.0103451
14	0.0765458	0.0300724	0.0300724	0.0122393	0.0122393	0.0140776	0.0140776
15	0.077912	0.0091016	0.0091016	0.0153514	0.0153514	0.0063838	0.0063838
16	0.0779638	0.0271572	0.0271572	0.0107977	0.0107977	0.0082761	0.0082761
17	0.0788466	0.0300598	0.0300598	0.0066013	0.0066012	0.0078798	0.0078798
18	0.0780435	0.0304332	0.0304332	0.0091368	0.0091368	0.0134464	0.0134464
19	0.0770202	0.0282309	0.0282309	0.0011616	0.0011616	0.0118429	0.0118429
20	0.0770612	0.0313027	0.0313027	0.0077819	0.0077819	0.0106729	0.0106729
21	0.0765107	0.0291399	0.0291399	0.0195865	0.0195865	0.0154014	0.0154014
22	0.0797588	0.0310287	0.0310287	0.0139931	0.0139931	0.0100695	0.0100695
23	0.0774463	0.0391174	0.0391174	0.0141666	0.0141666	0.0129255	0.0129255
24	0.0778448	0.0304949	0.0304949	0.007071	0.007071	0.0069126	0.0069126
25	0.0771923	0.0221736	0.0221736	0.0038151	0.0038151	0.0065912	0.0065912
26	0.0768698	0.0194513	0.0194513	-0.010136	-0.010136	0.0050403	0.0050403
27	0.0786199	0.0207589	0.0207589	0.004436	0.004436	0.0076542	0.0076542
28	0.0778698	0.0334576	0.0334576	0.0029954	0.0029954	0.0109504	0.0109504
29	0.0795129	0.0188308	0.0188308	0.0146876	0.0146876	0.0075499	0.0075499
30	0.0790069	0.0243548	0.0243548	0.0129958	0.0129959	0.0155163	0.0155163
Average	0.0779635	0.0250191	0.0250191	0.0094307	0.0094307	0.0094928	0.0094929

Supplementary Table 4 When filament density is 7.8%, the statistic numbers of Arp2/3 complex, filamin-A, α-actinin, crosslinking proteins (filamin-A + α-actinin) and actin filaments at the cross-section of y=1000, and the average length of actin filaments, r_a , in the RVE model.

NO.	V_f	Arp2/3	Filamin-A	α-actinin	Crosslinking proteins	Average length of filament, r_a	Number of actin filaments at y=1000
1	0.0778522	1559	1164	771	1935	0.2485194	319
2	0.0792666	1578	1076	670	1746	0.2496607	272
3	0.0771606	1526	941	560	1501	0.2509361	303
4	0.0780412	1553	1120	630	1750	0.2501986	277
5	0.0768723	1532	1146	669	1815	0.2498419	289
6	0.0771209	1541	1150	662	1812	0.2501807	328
7	0.0785634	1565	1157	662	1819	0.2492589	284
8	0.0787042	1563	1043	679	1722	0.2506234	284
9	0.0766642	1514	999	591	1590	0.2520023	291
10	0.078482	1562	1022	635	1657	0.2496101	329
11	0.0782078	1562	1056	682	1738	0.2491956	271
12	0.0786592	1551	1349	712	2061	0.2510958	337
13	0.0792858	1577	1192	729	1921	0.250328	294
14	0.0765458	1538	1121	628	1749	0.2476965	285
15	0.077912	1552	981	688	1669	0.2518485	270
16	0.0779638	1558	1123	662	1785	0.2513505	297
17	0.0788466	1544	1062	677	1739	0.2512433	278
18	0.0780435	1527	1004	682	1686	0.2533318	289
19	0.0770202	1529	904	669	1573	0.2493868	244
20	0.0770612	1545	1030	670	1700	0.248436	290
21	0.0765107	1512	1077	671	1748	0.2511801	299
22	0.0797588	1584	1015	717	1732	0.2501497	255
23	0.0774463	1522	936	576	1512	0.2518649	290
24	0.0778448	1551	1110	655	1765	0.249415	325
25	0.0771923	1541	987	647	1634	0.2474771	322
26	0.0768698	1528	1157	610	1767	0.2495217	283
27	0.0786199	1584	1168	639	1807	0.2477742	259
28	0.0778698	1538	1019	700	1719	0.2510429	243
29	0.0795129	1586	1112	723	1835	0.2495293	319
30	0.0790069	1591	1181	681	1862	0.2489937	362
Average	0.0779635	1550.4333	1080.0667	664.9	1744.9667	0.2500564	292.93333
Std.	0.0009176	21.623573	92.375658	44.242777	116.93374	0.0013584	27.32756

Supplementary Table 5 Comparison of Young's modulus E_2 in cell moving direction between our numerical simulation results with V_f from 3.0% to 9.8% and those from the *in vivo* and *in vitro* experiments.

Experiment type	Cell type	E_2 (kPa)	Actin filament density	Method	Ref.
Our prediction	--	0.6-39	3.0% ~ 9.8%	FEM numerical simulation	-
In vitro	--	1.0-20	--	Atomic force microscope (AFM)	ref.[9]
In vitro	--	0.8-5	--	AFM	ref.[2]
In vitro	--	1.0-10	--	Magnetic dipolar attraction between colloids	ref.[8]
In vivo	Adenocarcinoma cells	2.0-10	--	AFM	ref.[23]
In vivo	Fish epidermal keratocytes	21-44	about 8.0%	AFM	ref.[24]
In vivo	Motile 3T3 fibroblasts	3.0-12	--	AFM	ref.[25]

Supplementary References

1. Koestler SA, Rottner K, Lai F, Block J, Vinzenz M, Small JV. F- and G-actin concentrations in lamellipodia of moving cells. *PLoS One* **4**, e4810 (2009).

2. Chaudhuri O, Parekh SH, Fletcher DA. Reversible stress softening of actin networks. *Nature* **445**, 295 (2007).

3. Akin O, Mullins RD. Capping protein increases the rate of actin-based motility by promoting filament nucleation by the Arp2/3 complex. *Cell* **133**, 841-851 (2008).

4. Pollard TD, Blanchoin L, Mullins RD. Molecular mechanisms controlling actin filament dynamics in nonmuscle cells. *Annu Rev Biophys Biomol Struct* **29**, 545-576 (2000).

5. Abraham VC, Krishnamurthi V, Taylor DL, Lanni F. The actin-based nanomachine at the leading edge of migrating cells. *Biophys J* **77**, 1721-1732 (1999).

6. Maritzen T, Zech T, Schmidt MR, Krause E, Machesky LM, Haucke V. Gadkin negatively regulates cell spreading and motility via sequestration of the actin-nucleating ARP2/3 complex. *Proc Natl Acad Sci USA* **109**, 10382-10387 (2012).

7. Dang I, *et al.* Inhibitory signalling to the Arp2/3 complex steers cell migration. *Nature* **503**, 281 (2013).

8. Pujol T, du Roure O, Fermigier M, Heuvingh J. Impact of branching on the elasticity of actin networks. *Proc Natl Acad Sci USA* **109**, 10364-10369 (2012).

9. Bieling P, *et al*. Force Feedback Controls Motor Activity and Mechanical Properties of Self-Assembling Branched Actin Networks. *Cell* **164**, 115-127 (2016).

10. Blanchoin L, Boujemaa-Paterski R, Sykes C, Plastino J. Actin dynamics, architecture, and mechanics in cell motility. *Physiol Rev* **94**, 235-263 (2014).

11. Ridley AJ, *et al.* Cell migration: integrating signals from front to back. *Science* **302**, 1704-1709 (2003).

12. Svitkina TM, Borisy GG. Arp2/3 complex and actin depolymerizing factor/cofilin in dendritic organization and treadmilling of actin filament array in lamellipodia. *J Cell Biol* **145**, 1009-1026 (1999).

13. Medalia O, Weber I, Frangakis AS, Nicastro D, Gerisch G, Baumeister W. Macromolecular architecture in eukaryotic cells visualized by cryoelectron tomography. *Science* **298**, 1209-1213 (2002).

14. Flanagan LA, Chou J, Falet H, Neujahr R, Hartwig JH, Stossel TP. Filamin A, the Arp2/3 complex, and the morphology and function of cortical actin filaments in human melanoma cells. *J Cell Biol* **155**, 511-518 (2001).

15. Cameron LA, Giardini PA, Soo FS, Theriot JA. Secrets of actin-based motility revealed by a bacterial pathogen. *Nat Rev Mol Cell Biol* **1**, 110 (2000).

16. Ma Y, Zhu H, Su B, Hu G, Perks R. The elasto-plastic behaviour of three-dimensional stochastic fibre networks with cross-linkers. *J Mech Phys Solids* **110**, 155-172 (2018).

17. Ehrlicher AJ, Krishnan R, Guo M, Bidan CM, Weitz DA, Pollak MR. Alpha-actinin binding kinetics modulate cellular dynamics and force generation. *Proc Natl Acad Sci USA* **112**, 6619-6624 (2015).

18. Ahmed WW, Betz T. Dynamic cross-links tune the solid–fluid behavior of living cells. *Proc Natl Acad Sci USA* **112**, 6527-6528 (2015).

19. Broedersz CP, MacKintosh FC. Modeling semiflexible polymer networks. *Rev Mod Phys* **86**, 995 (2014).

20. Kojima H, Ishijima A, Yanagida T. Direct measurement of stiffness of single actin filaments with and without tropomyosin by in vitro nanomanipulation. *Proc Natl Acad Sci USA* **91**, 12962-12966 (1994).

21. Van Dillen T, Onck P, Van der Giessen E. Models for stiffening in cross-linked biopolymer networks: A comparative study. *J Mech Phys Solids* **56**, 2240-2264 (2008).

22. Wei X, Zhu Q, Qian J, Lin Y, Shenoy V. Response of biopolymer networks governed by the physical properties of cross-linking molecules. *Soft Matter* **12**, 2537-2541 (2016).

23. Rotsch C, Jacobson K, Condeelis J, Radmacher M. EGF-stimulated lamellipod extension in adenocarcinoma cells. *Ultramicroscopy* **86**, 97-106 (2001).

24. Laurent VM, *et al.* Gradient of rigidity in the lamellipodia of migrating cells revealed by atomic force microscopy. *Biophys J* **89**, 667-675 (2005).

25. Rotsch C, Jacobson K, Radmacher M. Dimensional and mechanical dynamics of active and stable edges in motile fibroblasts investigated by using atomic force microscopy. *Proc Natl Acad Sci USA* **96**, 921-926 (1999).
